# zDHHC-Mediated S-Palmitoylation in Skin Health and Its Targeting as a Treatment Perspective

**DOI:** 10.3390/ijms26041673

**Published:** 2025-02-15

**Authors:** Farah A. Abdulrahman, King A. Benford, Gregory T. Lin, Andrew J. Maroun, Caleb Sammons, Darya N. Shirzad, Harrison Tsai, Vincent L. Van Brunt, Zack Jones, Jafet E. Marquez, Evan C. Ratkus, Abdulrahman K. Shehadeh, Hugo Abasto Valle, Dea Fejzo, Ashlynn E. Gilbert, Catherine A. McWee, Lexie F. Underwood, Ethny Indico, Brittany B. Rork, Meera Nanjundan

**Affiliations:** Department of Molecular Biosciences, University of South Florida, 4202 East Fowler Avenue, ISA2015, Tampa, FL 33620, USA; abdulrahman20@usf.edu (F.A.A.); kbenford@usf.edu (K.A.B.); gtlin@usf.edu (G.T.L.); andrewjohnmaroun@usf.edu (A.J.M.); calebsammons@usf.edu (C.S.); daryashirzad@usf.edu (D.N.S.); harrisontsai@usf.edu (H.T.); vincentvanbrunt@usf.edu (V.L.V.B.); jones132@usf.edu (Z.J.); jemarquez14@usf.edu (J.E.M.); ecr1@usf.edu (E.C.R.); akshehadeh@usf.edu (A.K.S.); hugoa@usf.edu (H.A.V.); deafejzo@usf.edu (D.F.); aegilbert@usf.edu (A.E.G.); catherineannemcwee@usf.edu (C.A.M.); lexieunderwood@usf.edu (L.F.U.); ethny@usf.edu (E.I.); brittanybellerork@usf.edu (B.B.R.)

**Keywords:** skin, DHHC, S-palmitoylation, 2-bromopalmitate, phytochemicals, ErbB, PLSCR1, skin barrier function

## Abstract

S-acylation, which includes S-palmitoylation, is the only known reversible lipid-based post-translational protein modification. S-palmitoylation is mediated by palmitoyl acyltransferases (PATs), a family of 23 enzymes commonly referred to as zDHHCs, which catalyze the addition of palmitate to cysteine residues on specific target proteins. Aberrant S-palmitoylation events have been linked to the pathogenesis of multiple human diseases. While there have been advances in elucidating the molecular mechanisms underlying the pathogenesis of various skin conditions, there remain gaps in the knowledge, specifically with respect to the contribution of S-palmitoylation to the maintenance of skin barrier function. Towards this goal, we performed PubMed literature searches relevant to S-palmitoylation in skin to define current knowledge and areas that may benefit from further research studies. Furthermore, to identify alterations in gene products that are S-palmitoylated, we utilized bioinformatic tools such as SwissPalm and analyzed relevant data from publicly available databases such as cBioportal. Since the targeting of S-palmitoylated targets may offer an innovative treatment perspective, we surveyed small molecules inhibiting zDHHCs, including 2-bromopalmitate (2-BP) which is associated with off-target effects, and other targeting strategies. Collectively, our work aims to advance both basic and clinical research on skin barrier function with a focus on zDHHCs and relevant protein targets that may contribute to the pathogenesis of skin conditions such as atopic dermatitis, psoriasis, and skin cancers including melanoma.

## 1. Introduction

### 1.1. Overview of S-Palmitoylation and Its Cellular Importance

Protein S-palmitoylation is a post-translational modification (PTM) in which the attachment of a C16:0 palmitate occurs to a cysteine residue via a thioester linkage on a target protein [1]. While other forms of palmitoylation including O-palmitoylation and N-palmitoylation have been reported [2], S-acylation (of which S-palmitoylation is the most prevalent, in addition to unsaturated and saturated fatty acids such as arachidonate, stearate, and oleate [3]) is the only reversible lipid-based PTM [4]. This unique characteristic enables dynamic changes in protein trafficking and localization and protein stability, as well as protein interaction with lipid-rich microdomains within membranes [5,6]. The process of S-palmitoylation can occur on proteins that are either soluble or membrane-associated [7,8,9,10]. Since S-palmitoylation is a feature of proteins essential to the regulation of various cellular processes including signaling events [11], it is not surprising that the deregulation of these processes could contribute to the pathogenesis of various health conditions [12] including inflammation [13], metabolic diseases [14], and neurodegenerative diseases [15], as well as cancer [16]. Per Swiss Palm 2 (a database of protein palmitoylation sites), 10% of the human proteome is palmitoylated and the number of palmitoylated proteins is defined to be over 9000, encompassing 17 species [17].

### 1.2. Mechanism of S-Palmitoylation

**Figure 1:**
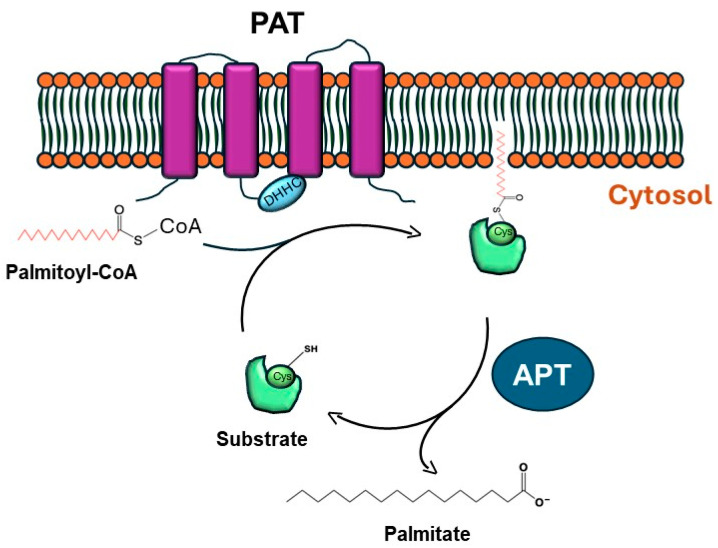
Mechanism of PAT-mediated protein substrate palmitoylation. The plasma membrane spanning palmitoyl acyltransferase (PAT) enzyme, characterized by a cytosolic DHHC motif, catalyzes the transfer of palmitate to a cysteine residue on target substrate proteins [1,18]. This is preceded by the autopalmitoylation of PAT on its DHHC motif which primes it for substrate modification [18]. This process is a reversible one, with acyl protein thioesterases (APTs) mediating the removal of the palmitate to restore the unmodified protein [18].

S-palmitoylation is regulated by two groups of enzymes (see Figure 1): (1) palmitoyl acyltransferases (PATs), which enzymatically catalyze the addition of palmitate to the protein target, and (2) acyl thioesterases (APTs), which enzymatically catalyze the removal of the palmitate moiety on the protein target [18]. The PAT mechanism of action is a two-fold enzymatic process: (a) auto-palmitoylation using palmitoyl-CoA of the zDHHC active site cysteine residue, followed by (b) the addition of the palmitate moiety to the protein target [18].

Human PATs are represented by a family of 23 enzymes known as zDHHCs, which contain a conserved Asp-His-His-Cys (DHHC) motif that is critical in supporting the palmitoylation event [19]. Although there are limited structural data for zDHHCs, the crystal structure of zDHHC20 is defined [20]. As depicted in [21], the zDHHC enzymes contain between 4 and 6 transmembrane regions; further, there is cytosolic exposure of the catalytic DHHC motif and cytosolic localization of both the N- and C-termini, with the exception of zDHHC4 and zDHHC24, whose C-termini are predicted to face the extracellular compartment [21]. In addition to the DHHC motif, specific zDHHC enzymes harbor other domains including ankyrin repeats in zDHHC13 and zDHHC17 [21,22], SH3 domains in zDHHC6 [21,23], PDZ domains in zDHHC5 and zDHHC8 [21,24], and ER retention signals in zDHHC4 and zDHHC6 [21,25].

While there exist an array of inhibitors targeting APTs, very few small molecules have been identified targeting zDHHCs. In particular, the well-studied inhibitor 2-bromopalmitate (2-BP) is associated with a multitude of off-target effects [26], which limits its clinical applicability. For this reason, our analyses focus on the current state of knowledge with respect to zDHHCs, specifically in skin health, along with perspectives on targeting strategies for PATs.

### 1.3. Skin Structure and Disruption of Skin Barrier Function in Skin Conditions

The most aesthetic and largest organ in humans is the skin, which functions as a protective barrier against mechanical stressors and microbial infections [27] while allowing environmental interactions and enabling regenerative responses to injuries [28,29]. From a developmental perspective, the ectoderm of the embryo gives rise to not only the skin but also the nervous system depending on extrinsic signaling events following the process of gastrulation [27]. Appendages that are associated with the skin include the hair follicles and sweat glands [27].

The skin organ is composed of the hypodermal layer, dermal layer, and epidermal layer [27]. The epidermis, from the bottom to the top-most layer, includes (a) the stratum basale which houses stem cells; (b) the stratum spinosum in which keratinocytes are bound to one another through desmosomes and adherens junctions (AJs); (c) the stratum granulosum in which cells (bound through both tight junctions (TJs) and AJs) contain filaggrin, which undergoes degradation to generate products eliciting hydration function, and lamellar glycolipid-filled granules—both components contribute to the maintenance of skin barrier integrity; (d) the stratum lucidum, specifically found in regions of increased skin thickness; and (e) the stratum corneum, which is composed of enucleated cornified keratinocytes, also called corneocytes [27,28]. In this outermost layer, these corneocytes are enveloped by lipids and within them, aggregated keratin is intertwined within a lipid network via envelope proteins [30,31]. These envelope proteins include small proline-rich proteins (SPRP), loricrin, involucrin, and filaggrin, which are cross-linked with one another via the activity of transglutaminase 1 (TGM1) [30,31]. The breakdown of filaggrin generates skin hydration factors, which are key in hindering microbial infections and maintaining skin barrier function [32].

Deregulation of any of the above-described components could potentially lead to the pathogenesis of skin conditions [33,34,35]. There exist a variety of skin disorders including acne vulgaris, alopecia areata, atopic dermatitis, epidermolysis bullosa, hidradenitis suppurativa, ichthyosis vulgaris, pemphigus vulgaris, psoriasis, rosacea, scleroderma, vitiligo, malignant melanoma, basal cell skin carcinoma, and cutaneous squamous cell carcinoma, amongst others (see Supplementary File S1, access date 8 September 2024). Current treatment regimens vary across these skin conditions, but commonalities include the application of topical ointments and/or emollients, antibiotics, topical steroids, and in some cases, surgery, radiation therapy, immunotherapy, photodynamic therapy, and/or chemotherapy [36,37,38,39,40,41,42,43,44,45,46,47,48,49]. A literature overview of the current state of the knowledge of these skin conditions in the context of palmitoylation or skin barrier function proteins identified atopic dermatitis and psoriasis with the highest number of hits (see Supplementary File S2, access date 7 July 2024), and these are therefore the focal points of our analyses.

### 1.4. Scope of Article

While there have been multiple advances in understanding the molecular mechanisms underlying skin development and disruption to skin barrier function, further investigations are needed for a comprehensive understanding of the contributing pathways. The purpose of the analyses presented herein is to advance both basic and clinical research on skin barrier function by identifying specific zDHHCs and protein targets that may contribute to the pathogenesis of skin conditions. To accomplish this, we performed PubMed literature searches, extracted and reviewed relevant data in publicly available databases, and utilized bioinformatic tools. The expression of zDHHC enzymes in skin cells and skin cancers was assessed using publicly available databases including cBioportal and Human Protein Atlas (HPA). Analyses of the literature pertaining to skin conditions and palmitoylation are also presented to define the current state of knowledge and identify gaps in the knowledge for future research investigations. Further to this, we analyze the literature pertaining to the current small molecules inhibiting zDHHCs and present future perspectives.

## 2. Literature Searches, Bioinformatic Tools, and Data Mining

### 2.1. Literature Searches Using PubMed

The date of each search term, the number of search results, and the specific search terms used were all recorded into an Excel spreadsheet. Those pertaining to (1) zDHHCs and intracellular trafficking search terms are shown in Supplementary File S3 (access date 29 June 2024); (2) 2-bromopalmitate, phytochemicals, and palmitoylation search terms are shown in Supplementary File S4 (access date 30 June 2024); (3) palmitoylation, skin diseases, and skin barrier function search terms are shown in Supplementary File S5 (access date 29 June 2024) and Supplementary File S6 (access date 2 July 2024); and (4) EGFR, scramblase, and palmitoylation search terms are shown in Supplementary File S7 (access date 29 June 2024).

zDHHCs, Intracellular Trafficking, and Signaling: The search terms used were “S-palmitoylation”, “zDHHC”, or “DHHC”, along with “skin” in combination with “human protein trafficking”, “actin”, “microtubule”, vimentin”, “lamin”, “keratin”, “kinesin”, “dynein”, “endoplasmic reticulum”, “Golgi”, “lysosome”, “autophagy”, “MAPK”, “AKT”, “PI3K”, “JAK”, “STAT”, “EGFR”, “GPCR”, and “subcellular”, from which a total of 34 unique articles were identified.

Palmitoylation, Skin Diseases, and Skin Barrier Function: The search terms used were “palmitoylation” or “DHHC” or “zDHHC” in combination with “skin”, “in vitro”, “in vivo”, “mouse”, “rat”, and 13 skin conditions including “acne”, “alopecia areata”, “atopic dermatitis”, “epidermolysis bullosa”, “hidradenitis suppurativa”, “ichthyosis”, “pachyonychia congenita”, “pemphigus”, “psoriasis”, “Raynaud’s phenomenon”, “rosacea”, “scleroderma”, and “vitiligo”, as well as 3 relevant cancer types, “melanoma”, “squamous cell”, and “basal cell”. In total, we identified 48 unique articles. Additional searches utilized search terms including “palmitoylation”, “DHHC”, or “zDHHC” along with proteins with major roles in supporting skin barrier such as “keratin”, “filaggrin”, “corneodesmosin”, “kallikrein”, “cathepsin”, “loricrin”, “involucrin”, “small protein-rich proteins”, “transglutaminase”, “cadherin”, “catenin”, “nectin”, “afadin”, “occludin”, “claudin”, “zona occludens”, “connexin”, “desmoglein”, “desmocollin”, “plakoglobin”, “plakophilin”, “envoplakin”, “periplakin”, and “plectin”, from which a total of 58 additional unique articles were identified.

EGFR and PLSCR: The search terms used were “ErbB1/EGFR”, “ErbB2”, “ErbB3”, and “ErbB4”, in combination with the terms “skin barrier function”, “palmitoylation”, and “palmitoylation skin/skin disease”. Likewise, similar searches were performed for the phospholipid scramblase family, which included “PLSCR1”, “PLSCR2”, “PLSCR3”, “PLSCR4”, “PLSCR5”, “XKR8”, and “TMEM16F”. Searches that combined “EGFR and PLSCR1” were additionally performed. In total, across these searches, 81 unique articles were identified.

2-Bromopalmitate, Phytochemicals, and Palmitoylation: The search terms used included “2-bromopalmitate”, “cerulenin”, “tunicamycin”, “cyano-myracrylamide”, “curcumin”, “artemisinin”, “ketoconazole”, “bis-piperazine”, “lanatoside C”, and N-cyanomethyl-N-myracrylamide”. In total, across the performed searches, 49 unique articles were identified.

For all relevant hits identified in the above searches, full-length PDF articles were obtained for further analysis.

### 2.2. Bioinformatic Tools

SwissPalm is a database for protein S-palmitoylation that was accessed through https://swisspalm.org/ (Version 4, 2022) [50]. The access date was 17 September 2024 for Table 1 and 1 September 2024 for Table 2. Searches were performed based on protein names for the human forms of zDHHCs, skin barrier function proteins, the ErbB, and PLSCR families. The data that were extracted include the UniProt ID#, cysteine residue sites, and experimentally validated S-palmitoylation sites.

Expasy is a Swiss bioinformatics resource portal that was accessed through https://www.expasy.org/ 25 August 2024 [51,52]. This site includes UniProt (https://www.uniprot.org, accessed on 25 August 2024) and UniProt Align (https://www.uniprot.org/align, 25 August 2024), which were utilized to extract the size (kDa) and amino acid number of specific proteins. The access date was 25 August 2024 for both Table 1 and Supplementary File S11, and 31 August 2024 for Table 2. Ensembl, accessed through https://useast.ensembl.org/index.html (accessed on 25 August 2024) (Version Release 112, May 2024), is a vertebrate genome online browser [53]. It was utilized to extract information on the 23 human zDHHC enzymes including their chromosomal location, base pair length, and protein amino acid length. The protein size for zDHHCs (in kDa) was obtained through UniProt, linked directly from Ensembl. The access date was 25 August 2024 for both Table 1 and Supplementary File S11, and 31 August 2024 for Table 2. HUGO Gene Nomenclature (HGNC) is an online resource for approved gene names, gene symbols, and chromosomal locations that was accessed through https://www.genenames.org [54]. The access date was 25 August 2024 for Table 1 and Supplementary File S11, and 31 August 2024 for Table 2.

### 2.3. Data Mining

The Human Protein Atlas (HPA) is a protein curation database that was accessed through https://www.proteinatlas.org/ (Version 23.0) [55,56,57,58,59,60,61]. This database integrates current human transcriptomic and protein expression data that are derived from the integration of various OMICS technologies (e.g., antibody-based histochemistry, MS-based proteomics, RNA profiling, and systems biology) across major human tissues/organ systems and cell types. Our investigation focused on extracting expression data at both the mRNA and protein level for zDHHC enzymes relevant to skin tissue. Moreover, we documented the availability of antibody tools towards zDHHCs and the sensitivity of methodologies applied to obtain their expression profiling data in order to uncover potential improvement areas for future experimental tool development. The access date was 22 November 2024).

cBioportal is a publicly available online tool for the analysis of genomic data pertaining to various cancer types that was accessed through https://www.cbioportal.org accessed on 16 November 2024 [62,63,64]. The combined study for cancers relevant to the skin included 3777 samples (across 3663 patients) from 21 studies including (a) acral melanoma (TGEN, Genome Res 2017) [65], (b) basal cell carcinoma (UNIGE, Nat Genet 2016) [66], (c) cutaneous melanoma (TCGA, GDC) [Source data from GDC and generated in July 2024 using ISB-CGC BigQuery tables (https://isb-cgc.appspot.com/bq_meta_search/, accessed on 16 November 2024)], (d) cutaneous squamous cell carcinoma (DFCI, Clin Cancer Res 2015) [67], (e) cutaneous squamous cell carcinoma (MD Anderson, Clin Cancer Res 2014) [68], (f) cutaneous squamous cell carcinoma (UCSF, NPJ Genom Med 2021) [69], (g) cutaneous squamous cell carcinoma (UOW, Front Oncol 2022) [70], (h) desmoplastic melanoma (Broad Institute, Nat Genet 2015) [71], (i) melanoma (Broad/Dana Farber, Nature 2012) [72], (j) melanoma (MSK, Clin Cancer Res 2021) [73], (k) melanoma (MSK, NEJM 2014) [74], (l) melanomas (TCGA, Cell 2015) [75], (m) metastatic melanoma (DFCI, Nature Medicine, 2019) [76], (n) metastatic melanoma (DFCI, Science 2015) [77], (o) metastatic melanoma (MSK, JCO Precis Oncol 2017) [78], (p) metastatic melanoma (UCLA, Cell 2016) [79], (q) skin cutaneous melanoma (Broad, Cell 2012) [80], (r) skin cutaneous melanoma (TCGA, Firehose Legacy) [Source data from GDAC Firehose (https://gdac.broadinstitute.org/runs/stddata__2016_01_28/data/SKCM/20160128/, accessed on 16 November 2024. Previously known as TCGA Provisional], (s) skin cutaneous melanoma (TCGA, PanCancer Atlas) [81,82,83,84,85,86,87,88,89], (t) skin cutaneous melanoma (Yale, Nat Genet 2012) [90], and (u) skin cutaneous melanoma (Broad, Cancer Discov 2014) [91]. We focused our analyses on genes relevant to skin barrier function and zDHHCs, as well as EGFR and scramblase family members. The access date was 16 November 2024 (refer to Supplementary Files S8–S10).

## 3. S-Palmitoylation and Skin Health

### 3.1. Chromosomal Location of zDHHCs and Skin Barrier Function Proteins: Proximity to Susceptibility Gene Loci in Atopic Dermatitis and Psoriasis

Atopic dermatitis, a form of eczema, is an inflammatory condition of the skin [38]. The pathogenesis of this condition is multifactorial including both genetic and environmental components [38]. Hallmarks of this condition include defects in skin barrier function (e.g., loss of functional filaggrin and reduction in lipids in the cornified envelope) leading to loss of hydration, pruritus, and subsequently, increased skin penetration of microbes and other irritants leading to inflammation [38]. Another inflammatory skin condition is psoriasis. Hallmarks of psoriasis include red-colored plaques that are thickened due to epidermal hyperkeratosis and skin dryness [43]. Susceptible gene loci have been identified as genetic determinants in atopic dermatitis as well as psoriasis from genome-wide association studies (GWASs) and are reviewed in [92,93].

To determine whether these GWAS locus sites were located within or in close proximity to regions where zDHHCs are genomically positioned, we summarize their genomic locations in Table 1 and Supplementary File S11 based on data extracted from HGNC [54].

While none of the GWAS locus mapped genes are zDHHCs in themselves in atopic dermatitis, we note that several of the zDHHCs are located within or in nearby GWAS sites, including zDHHC4, zDHHC12, zDHHC13, zDHHC18, zDHHC23, and zDHHC24. In psoriasis, several zDHHCs are located within or nearby GWAS sites, including zDHHC8, zDHHC13, zDHHC14, zDHHC16, zDHHC18, zDHHC20, and zDHHC24. To assess whether the reported GWAS locus sites are also located within or in close proximity to the chromosomal locations of skin barrier function proteins, we summarize their locations in Table 2. We note that several of them are located within or in nearby GWAS sites in atopic dermatitis, including claudin-6, nectins, desmosomal plaque proteins (i.e., plakophilins, plakoglobin, periplakin, desmogleins, and desmocollins), plectin, corneodesmosin, and envelope proteins (i.e., filaggrin, keratinocyte proline-rich protein, corniferin, small proline-rich proteins, involucrin, loricrin, and PADi3). In psoriasis, several skin barrier function proteins are located within or nearby GWAS sites including claudin-5, claudin-6, claudin-14, zonula occludens-1 (ZO-1), connexin 26 (Cx26), nectins, desmosomal plaque proteins (i.e., plakophilins, plakoglobin, envoplakin, and periplakin), and envelope proteins (i.e., filaggrin, keratinocyte proline-rich protein, corniferin, small proline-rich proteins, involucrin, loricrin, and PADi3). The significance of these findings to the pathogenesis of these conditions remains unclear and requires further investigation.

### 3.2. Methodologies Applied in the Study of Palmitoylation with Respect to Skin Health

Methodologies that have been applied in the study of palmitoylation, specifically in the context of skin health, are summarized in Table 3 and Supplementary File S12. These methods include (1) metabolic labeling using [1-^14^C]-palmitic acid, (2) metabolic labeling using [^3^H]-palmitic acid, (3) Matrix-Assisted Laser Desorption/Ionization Time-of-Flight Mass Spectrometry (MALDI-TOF-MS), (4) immunoprecipitation (IP) followed by acyl-biotin exchange (ABE), (5) metabolic labeling using 17-octadecynoic acid (17-ODYA) followed by ABE, (6) cell labeling using palmitic acid azide followed by click chemistry using biotin alkyne (Click-iT assay), (7) the ABE to MS approach, (10) the alkylating resin-assisted capture (RAC) assay, and (11) cell labeling using Alk-14 followed by click chemistry for in-gel fluorescence. As shown in Table 3 and Supplementary File S12, methods to predict palmitoylation sites include the use of publicly available online tools such as CSS-Palm (Version 2.0, 3.0, or 4.0), which have uncovered predicted cysteine residues that may acquire palmitates on claudin-1, claudin-3, and claudin-4 [94], claudin-5 [95], claudin-2, Cx32, and ZO-1 [96] as well as tyrosinase [97].

Experimental methods including metabolic labeling with [1-^14^C]-palmitic acid were applied to assess whether TGM1 was palmitoylated in normal human epidermal keratinocytes (NHEKs) [98] and in keratinocytes [99]. Metabolic labeling using [^3^H]-palmitic acid was applied to assess the palmitoylation status of TGM1 in NHEK cells [98]; Gsa in A431/μ cells [100]; claudin-14 in MDCK cells [101]; occludin, claudin-1, and claudin-2 in MDCK II cells [102]; zDHHC21 substrates (eNOS and Lck) in HEK293 cells [103]; integrin β4 in HEK293 cells [104]; melanoregulin in RPE and Cos7 cells [105]; and myc-CDC42 variants in human brain microvascular endothelial cells (HBMECs) [106].

ABE was utilized more widely to determine the palmitoylation status of myc-tagged huntingtin from HEK293T cells [107]; β-catenin in HEK293T cells [108] and PTC cells [109]; δ-catenin in glycine/bicuculline-treated hippocampal neurons [110] and in spinal dorsal horn/DRG tissues treated with oxaliplatin [111], as well as in CWR22Rv1 and PC3 cells [112]; desmosomal plaque proteins and cadherins in A431 cells [113]; CD44 and Cav-1 in MCF10A cells [114]; claudin-7 in HEK293 cells [115] or in ASML cells [116]; myc-tagged cornifelin in HEK293T cells [117]; skin proteins in wild-type and zDHHC13 mutant mice [118]; zDHHC5 in hippocampal neurons [119]; desmoglein-2 in A431 cells [120]; MC1R in HPM-RHC cells [121] and HPM cells [122]; Drp1 in murine cerebella from wild-type and mutant zDHHC13 mice [123]; loricrin, PADi3, and TGM1 in skin derived from wild-type and mutant zDHHC13 mice [124]; Cx32 in LNCaP cells [125]; and protocadherin 7 (PCDH7) in HeLa S3 cells [126]. Another recent methodological advancement includes the application of the alkylating resin-assisted capture (RAC) method to determine the S-palmitoylome in the liver from wild-type and zDHHC13-deficient [127] or mutant mice [124] and to assay for tyrosinase palmitoylation in HM3KO cells [97].

The application of proteomic approaches was also applied including MALDI-TOF-MS to assess the palmitoylation status of Cx26 and Cx32 purified from mouse tissue [128] and MS which was applied for determination of the palmitoylation levels of purified claudin-3, claudin-4, and claudin-6 from HEK-293 cells [129]. The combination of ABE with MS proteomics was applied to assay the global palmitoylation profile in skin proteins from wild-type and zDHHC13 mutant mice [117]. In some cases, ABE was utilized after metabolic labeling with 17-ODYA to assess the palmitoylation status of plakophilins in A431 cells [113] or followed by a click chemistry approach to determine the S-palmitoylome in normal and fibrotic murine kidneys [109]. Click chemistry was sometimes utilized alone (using Click-iT palmitic acid with azide) to assess the palmitoylation of β-catenin in PTC cells [109], after metabolic labeling with 17-ODYA to assay for the tyrosinase palmitoylation state in HM3KO cells [97], and after cell labeling with palmitic acid azide to assay for Fas palmitoylation in HEK293T cells [130], as well as MCAM and CD44 in WM239A melanoma cells [114], or following cell labeling with Alk-14 to assess the palmitoylation state of FLAG-tagged claudin-3 in HEK293T cells [131].

As noted from Table 3 and Supplementary File S12, prior to 2015, the common palmitoylation methodology that was applied was metabolic labeling using radiolabeled palmitic acid. This appears to have now transitioned predominantly to the application of ABE methodology. We direct the reader to published comprehensive reviews for further details with regards to the above-described methods [132].

### 3.3. Palmitoylation Status of zDHHCs and Skin Barrier Function Proteins

Per the review of SwissPalm, the majority of zDHHCs were predicted to contain palmitoylation sites. However, only zDHHC6 was experimentally demonstrated to be palmitoylated. The palmitoylation sites on zDHHC6 were defined as Cys328, Cys329, and Cys343, located within its SH3 domain, which was dependent on zDHHC16 and mediated via a direct protein–protein interaction [23].

With regards to skin barrier proteins, we identified predicted palmitoylated sites through SwissPalm for the human forms (Table 2). Further, per our review of the literature using defined search terms, we noted that a subset of proteins involved in skin barrier function are palmitoylated, along with the identification of specific palmitoylation sites (Table 4 and Supplementary File S13).

Common palmitoylation methods including metabolic labeling with either [1-^14^C]-palmitic acid or [^3^H]-palmitic acid, ABE, metabolic labeling with 17-ODYA or Alk-14 followed by click chemistry, ABE proteomics, or alkylating RAC; or native mass spectrometry were utilized to define the palmitoylation status of the skin barrier function proteins. These studies predominantly utilized common laboratory cell lines (e.g., HEK293T, HEK293, and COS7) but, in some cases, MDCK II, NHEK, and A431 which are relevant as epidermal cell line models were also utilized (Table 4 and Supplementary File S13). Largely, many of the skin barrier function proteins that were investigated (the majority of which are human or murine forms) were experimentally shown to be palmitoylated, using our defined literature search terms. The specific palmitoylation sites, when investigated in specific cases, were predicted using CSS-Palm and in some cases, sites were confirmed via mutagenesis studies in which cysteine sites were mutated.

*ErbB Family Members:* We have included EGFR in our analyses, which plays a critical role in supporting skin barrier integrity and has been experimentally demonstrated to be palmitoylated (see Supplementary File S14).

While EGFR, also known as ErbB1, is palmitoylated [133], we extended the analyses to other ErbB family members and to one interacting partner, phospholipid scramblase 1 (PLSCR1, and its family members) which is also palmitoylated [134]. While other ErbB members were predicted to be palmitoylated via SwissPalm, we did not uncover any experimental support of their palmitoylation status using our specific search terms (Table 5). Altogether, these analyses suggest that palmitoylation may be a critical event to support the integrity of the skin barrier function.

*Claudins:* Endogenous canine claudin-1 in MDCK II cells was experimentally shown to be palmitoylated [102], while the human form was predicted to be palmitoylated between transmembrane domain 2 and 3 and in its cytoplasmic tail (at Cys104, Cys107, Cys183, Cys184, and Cys186) [94]. Both endogenous canine [102] and the overexpressed human form of claudin-2 [101] were also experimentally shown to be palmitoylated, although no cysteine palmitoylation sites were predicted in the human form [96]. Not only was human claudin-3 predicted to be palmitoylated between transmembrane domain 2 and 3 as well as its cytoplasmic tail (at Cys103, Cys106, Cys181, Cys182, and Cys184) [94], experimental results are available that support its palmitoylation state [129,131]. Human claudin-4 was similarly predicted to be palmitoylated between transmembrane domain 2 and 3 as well as in its cytoplasmic domain (at Cys104, Cys107, and Cys183) [94]; further, it was experimentally demonstrated to be palmitoylated [129]. While human claudin-5 was only predicted to be palmitoylated (at Cys104, Cys107, Cys182, and Cys183) [95], human claudin-6 [129], the human and rat forms of claudin-7 [115,116], and human claudin-14 [101] were all experimentally demonstrated to be palmitoylated.

*Catenin:* Prediction, as well as experimental evidence, supports the palmitoylation status of human β-catenin [108,109]. For δ-catenin, experimental findings support the palmitoylation status of the human [110,112], murine [112,127], and rat [111] forms.

*Connexin*: While murine Cx26 and rat Cx32 were not identified to be palmitoylated in one study [128], an independent report provided experimental support for the palmitoylation of rat Cx32 [125]. In addition, murine Cx32 was experimentally demonstrated to be palmitoylated [128]. Human Cx32 was predicted to be palmitoylated at Cys280 and Cys283 [96].

*Desmosomal Plaque Proteins:* The desmosomal cadherins, namely human desmoglein-2 [113,120,135,136], murine desmoglein-3 [137], human desmoglein-3, and desmocollin-2 [113], were demonstrated experimentally to be palmitoylated. Moreover, the desmosomal plaque proteins, namely human plakophilin-2, human plakophilin-3, and human plakoglobin, were also experimentally shown to be palmitoylated [113].

*Envelope Proteins:* Experimental data support the palmitoylation status of endogenous human TGM1 [98], overexpressed TGM1 [99], drosophila TGMA [138], and murine TGM1 [124]. In other reports, TGM1 was only described as being palmitoylated or amino acid residues specified (Cys47, Cys48, Cys50, and Cys51) but no experimental data were shown [139,140]. Envelope proteins demonstrated to be palmitoylated using a zDHHC13-deficient mouse model include filaggrin-2, keratinocyte proline-rich protein, cornifin-A/B, small proline-rich protein 2D/2I/2E, involucrin, loricrin, and PADi3 [124].

*Other Skin Barrier Function Proteins:* Although zonula occludens 1 (ZO-1) is predicted to be palmitoylated at Cys744, Cys1718, and Cys1740 [96], no supporting experimental literature was uncovered using our search terms. Occludin, which is an important tight junctional component along with claudins, was demonstrated to lack palmitoylation [102]. Furthermore, no data were uncovered to experimentally support the palmitoylation status of nectins [141]. However, human integrin α6β4, a critical mediator of cell adhesion to the basement membrane, was experimentally shown to be palmitoylated [104].

### 3.4. Contribution of zDHHCs to Skin Health

The following describes the current literature supporting roles for zDHHCs in skin health using mouse models as the main experimental approach.

**Figure 2:**
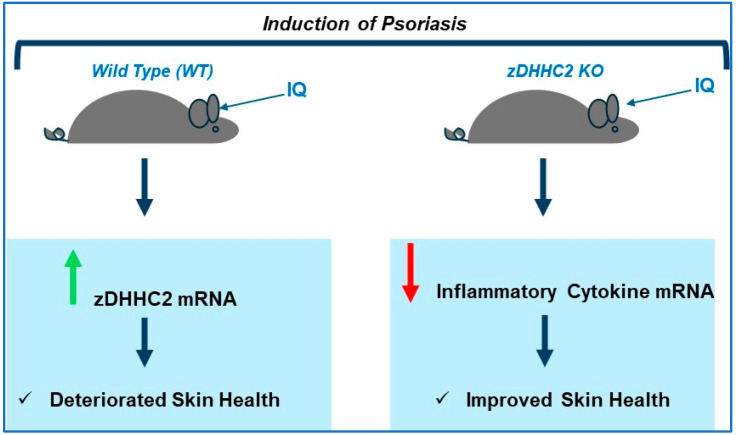
Imiquimod (IQ) induces psoriasis-like inflammation in wild-type (WT) and zDHHC2 knockout (KO) mice. In wild-type (WT) mice, the expression of zDHHC2 mRNA increases (depicted by vertical green arrow), which is associated with deteriorated skin health. In contrast, zDHHC2 knockout (KO) mice elicited reduced (depicted by vertical red arrow) inflammatory cytokine mRNA expression, which was associated with improved skin health [142].

*zDHHC2 Knockout Murine Model:* A summary is presented in Figure 2 below.

Imiquimod (IQ) treatment of C57BL/6 murine ears, which induces the pathogenesis of psoriasis, was associated with increased zDHHC2 transcript expression [142]. A zDHHC2 deficiency in the mouse (generated through the use of a CRISPR/Cas9 approach) improved the health of the ear skin (i.e., reduced erythema, scaling, and thickness) when imiquimod was applied (for 8 days) compared to wild-type mice [142]. Other notable improvements that accompanied zDHHC2 deficiency include reduced inflammatory response as assessed via transcript measurements of pro-inflammatory cytokines including IFN-α, TNF-α, IL-23, and IL-17A [142].

**Figure 3:**
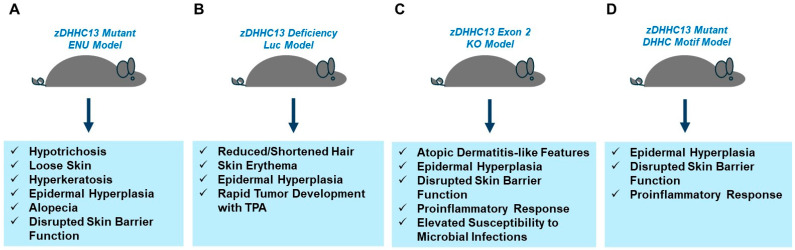
Murine models of zDHHC13 and alterations in skin health. The murine models include (**A**) zDHHC13 ENU mutant [107,117], (**B**) zDHHC13-deficiency Luc model [118], (**C**) zDHHC13 knockout (exon 2) model [145], and (**D**) zDHHC13 DHHC motif mutant [124].

*zDHHC13 Mutant/Deficient Murine Models:* A summary is presented in Figure 3 below.

Mutant zDHHC13 mice were identified through an N-ethyl-*N*-nitrosourea-induced (ENU) mutagenesis screening approach [107]. These mice were characterized by hypotrichosis and wrinkled/loose skin, amongst other features including reduced weight, reduced lifespan, amyloidosis, and osteoporosis [107]. Specifically, histopathological analyses of the skin uncovered hyperkeratosis and epidermal hyperplasia along with reduced active hair follicles [107]. Along with the above skin abnormalities in the mutant zDHHC13 mice, further examination revealed a dry and dull hair quality, “ragged” hair shafts, complete alopecia by day 27, increased hair release, and increased cornified layers within the hyperkeratosis regions [117]. The stratum corneum had increased fragility in addition to disrupted skin barrier function [117]. Through an ABE-proteomic approach to assay for global palmitoylation, alterations in the palmitoylation of targets involved in skin health were identified [117]. One target included cornifelin, a cornified envelope protein, which was not palmitoylated in the zDHHC13 mutant mice; further, the protein abundance was reduced [117]. Cornifelin was also confirmed to be a target of zDHHC13 in HEK293T cells [117]. Further examination of the skin and hair appendage of the zDHHC13 mice at 4 weeks of age uncovered that cornifelin was not detectable at the protein level [117], which suggests that it may contribute to the observed phenotypic alterations in the mutant zDHHC13 skin and appendages [117]. In an independent study, the S-palmitoylome of livers from ENU-induced zDHHC13 mutant mice was assessed using an alkyl-RAC palmitoylation methodology [127]. Many of the identified targets were categorized into those regulating lipid metabolism and mitochondrial function in the mutant mice [127]. With evidence that mitochondria are key mediators of skin [143] as well as hair health [144], these palmitoylation findings, though in the context of the liver, are therefore likely to be highly relevant to aspects of skin health as well.

Further evidence of the contribution of the loss of zDHHC13 to mitochondrial function arises from in vivo studies using a *luc* mouse model that is deficient in zDHHC13, which is characterized by reduced S-palmitoylation on dynamin-related protein 1 (Drp1, a mitochondrial fission protein that is a defined substrate of zDHHC13), in the context of bioenergetics in brain tissue as well as anxiety-induced behavioral changes [123]. This *luc* mouse model contains a mutation in the zDHHC13 gene that is associated with reduced PAT transcript expression and a lack of PAT activity [118,123]. These zDHHC13-deficient mice also displayed abnormalities in their skin including reduced/shortened hairs, skin erythema, and epidermal hyperplasia [118], in the absence of other characteristics noted with the ENU-induced zDHHC13 mouse model [107]. Interestingly, isolated keratinocytes from the mutant mice elicited increased migratory potential in a wound healing assay [118]. The increased proliferative capacity of the epidermis in the mutant mice was also noted with UVB treatment along with activated STAT3, p21, and p53 proteins [118]. Since tumor development was also more rapid in the mutant mice following TPA (12-O-tetradecanoylphorbol-13-acetate) treatment [118], this suggests that zDHHC13 may elicit a protective function against skin cancer development [118].

The phenotype of a knockout zDHHC13 mouse (with a deletion of exon 2 producing an early termination product of 13 amino acids) [145] was reported to be similar to that described for the ENU-induced mutagenesis zDHHC13 mouse model [107]. Specifically, the features of this knockout mouse model include atopic dermatitis-like characteristics, thickening of the skin, and elevated pro-inflammatory cytokines in the skin (e.g., IL-1β and TNF-α) [145]. In a keratinocyte-specific zDHHC13-deficient mouse model, the infiltration of immune cells, increased epidermal proliferation, and altered hair quality were also noted [145]. Further, skin barrier integrity was defective in the zDHHC13-deficient mice, as determined using a permeable toluidine blue dye, which was accompanied by altered processing of filaggrin [145]. Interestingly, the application of an antibiotic ointment to the ENU-induced zDHHC13 mice hindered keratinocyte proliferation and the loss of hair, implicating the susceptibility of these mice to microbial infections [145].

A transgenic mouse model in which zDHHC13 harbors a mutant DHHC motif (i.e., AAHC) resulting in a palmitoyltransferase dead form was produced to assess whether PAT activity contributes to the aforementioned alterations in skin health and the detrimental effect on skin barrier function [124]. The phenotype of these mutant mice appeared to be similar to the knockout zDHHC13 mice and ENU-induced mutagenesis mice including disrupted skin barrier function, increased hyperplasia of the epidermis, mast cell infiltration, pro-inflammatory response, and reduced degradation of filaggrin [124]. Using RAC-labeling and a proteomics approach, more than 3000 S-palmitoylation sites across 1278 proteins in skin specimens were identified and the allergy pathways (followed by pathways linked to psoriasis and atopic dermatitis) were defined as the most significantly altered through Ingenuity Pathway Analyses (IPA) [124]. Within this dataset, 14 relevant skin barrier function proteins were identified with reduced palmitoylation levels which included PADi3, TGMase 1, and loricrin [124]. These latter proteins were validated through co-immunoprecipitation and ABE assays [124]. Additionally, the protein abundance of PADi3 and TGMase 1 were markedly reduced [124].

**Figure 4:**
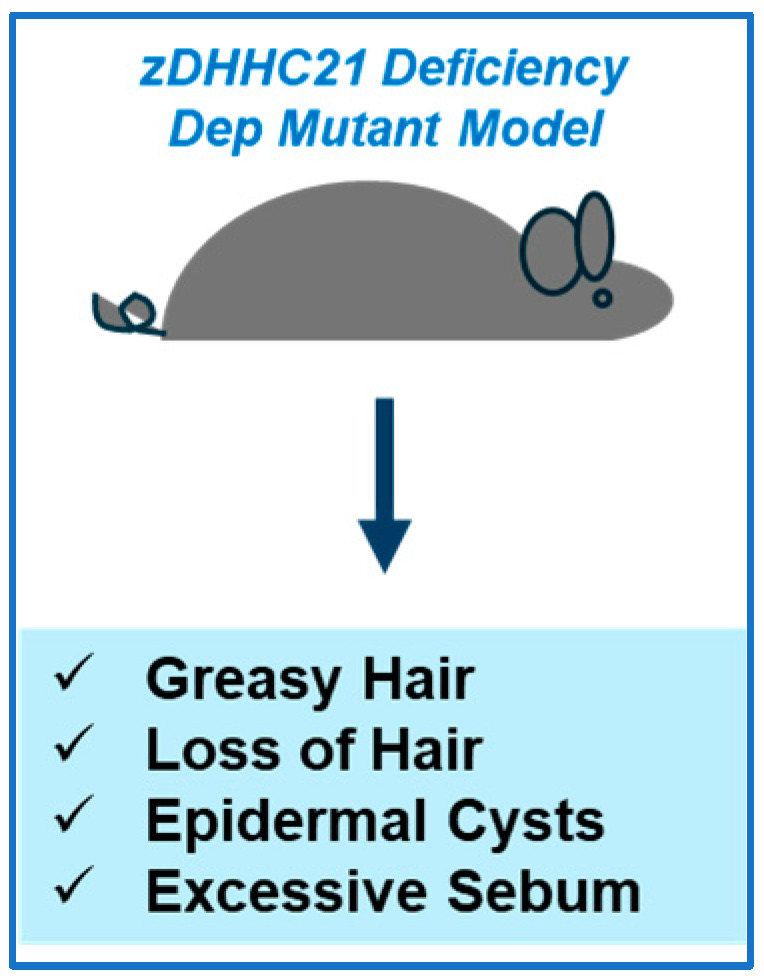
zDHHC21 deficiency and alterations in skin health. Deficiency of zDHHC21 was identified in *Dep* mutant mice and was associated with changes in the skin health and its associated hair appendage [103].

*zDHHC21 Mutant Murine Models:* A summary is presented in Figure 4 below.

*Dep* mutant mice, characterized by greasy hair as well as a loss of hair, were found to contain a deletion in the *zDHHC21* gene within the affected genomic interval [103]. The greasy hair feature appears to be attributed to large sebaceous glands along with excessive sebum and epidermal cysts in some cases [103]. The signaling pathways (i.e., MAPK, β-catenin) involved in the maintenance of the epidermis were found to be affected by loss of zDHHC21 [103]. zDHHC21 expression was also found to be localized to the follicular inner root sheathes [103]. Not only was the mutated zDHHC21 mislocalized to the cis-Golgi rather than the expected endoplasmic reticulum compartment when expressed in keratinocytes and NIH 3T3 cells, but it lacked palmitoyltransferase activity towards eNOS and Lck substrates when the cells were metabolically radiolabeled with [^3^H]-palmitate [103].

### 3.5. Contribution of Palmitoylation of Melanocortin-1 Receptor to Melanomagenesis

**Figure 5:**
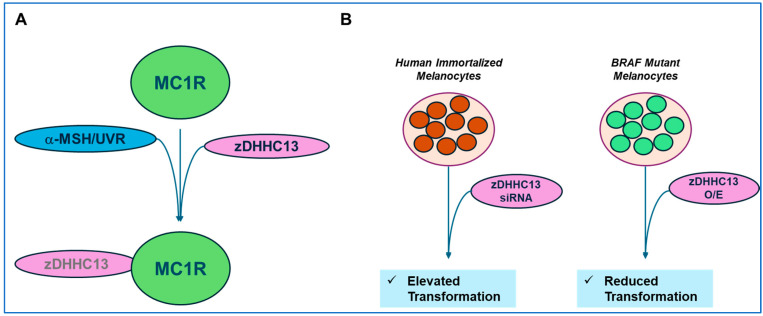
Role of zDHHC13 role in MC1R signaling in melanocytes. (**A**) Melanin production by *α*-MSH along with ultraviolet radiation (UVR) causes zDHHC13 association with MC1R [121]. (**B**) In human immortalized melanocytes, zDHHC13 (siRNA) knockdown increases transformation [121] while zDHHC13 overexpression (O/E) in BRAF mutant melanocytes reduces the transformative response [121].

*Melanocortin-1 (MC1R) and Melanomagenesis:* A summary is presented in Figure 5 below.

MC1R is a G-protein coupled receptor (GPCR) that is responsible for pigmentation, and its activation via melanocortin stimulating hormone (α-MSH) leads to the production of melanin as well as protection against DNA damaging events induced by ultraviolet radiation (UVR) that may contribute to the pathogenesis of melanoma [121]. It is well established that red hair color (RHC) individuals contain variants of MC1R that are associated with an elevated risk of skin damage progressing to melanoma [121]. In a murine melanoma B16 cell line, both endogenous and exogenous wild-type MC1R were demonstrated to be palmitoylated (at Cys315) upon activation [121]. In contrast, the MC1R R151C and R160W mutants elicited reduced palmitoylation following UVR treatment [121]. While several overexpressed zDHHC members, namely zDHHC2, zDHHC3, zDHHC7, zDHHC13, and zDHHC17, could each palmitoylate MC1R, siRNA targeting zDHHC13 reduced MC1R palmitoylation effectively [121]. A direct interaction between zDHHC13 and wild-type MC1R (and reduced binding with the mutants) was demonstrated via co-immunoprecipitation in cells treated with α-MSH and UVR [121]. Interestingly, a reduction in zDHHC13 promoted cellular transformation in human immortalized melanocytes [121] while the overexpression of zDHHC13 diminished the transformative response when using BRAF mutant melanocytes in a murine subcutaneous xenograft study [121]. However, the progeny generated by crossing MC1R loss-of function mice with re-expressed MC1R variants to BRAF mutant mice were characterized by the early development of melanomas following weekly UVR treatment (up to 4 weeks) [121]. From a TCGA analysis as well as GEPIA (“Gene Expression Profiling Interactive Analysis”), an association was uncovered between the transcript abundance of MC1R and the expression of downstream targets (i.e., MITF); furthermore, zDHHC13 itself was correlated with increased survival [146]. A melanocyte-specific zDHHC13 transgenic mouse model was crossed with mice expressing the MC1R R151C variant or MC1R C315S variant (non-palmitoylated) [146]; this model demonstrated that the palmitoylation level of MC1R R151C (as determined via ABE) was markedly increased, suggesting that zDHHC13 can reverse susceptibility to UVB-induced melanoma progression [146]. Moreover, when the MC1R C315S mouse model was crossed with the melanocyte-specific zDHHC13 transgenic mouse model, UVB-induced melanoma was detected sooner relative to the MC1R R151C mice followed by the wild-type mice [146].

Palm-B, a small molecule inhibitor of diacylation, leads to increased levels of palmitoylated wild-type and R151C MC1R proteins in B16 cells [121]. However, since the palmitoylation of many targets is likely increased with Palm-B, targeting zDHHC13 is suggested to be a better option in preventing melanoma development; therefore, investigating mechanisms underlying its regulation may offer additional therapeutic strategies. In this regard, a new Ser208 phosphorylation site on zDHHC13 was defined within its ankyrin domain [122]. This site is phosphorylated by AMPK-α, which directly interacts with zDHHC13 [122]. One consequence of zDHHC13 phosphorylation is MC1R palmitoylation at Cys315, which becomes reduced by the knockdown of AMPK-α or treatment with Compound C, an AMPK inhibitor [122]. Functionally, using a melanoma mouse model, AMPK-α activation is correlated with protective effects in UVB-mediated melanomagenesis [122].

### 3.6. Contribution of Palmitoylation of Tyrosinase to Melanogenesis

*Tyrosinase and Melanogenesis:* Melanocytes in the mammalian system generate two types of melanin: (a) black eumelanin and (b) yellow-red pheomelanin [147]. The three-enzyme-based system is comprised of tyrosinase (the rate-limiting enzyme) as well as tyrosinase-related protein 1 (TRYP-1) and tyrosinase-related protein 2 (TRYP-2), which are involved in the later stages of eumelanin generation [148]. With respect to the role of tyrosinase palmitoylation in melanogenesis, 2-BP (25 μM, 48 h) treatment in HM3KO human melanoma cells reduced its palmitoylation level but increased protein levels (implicating elevated protein stability), as determined by the acyl-RAC and 17-ODYA methods [97]. A C500A mutant failed to acquire palmitoyl moieties, indicating that the C500 residue is critical in tyrosinase palmitoylation [97]. Several zDHHC enzymes contributed to tyrosinase palmitoylation in HEK293T cells including zDHHC2, zDHHC3, zDHHC7, and zDHHC15; likewise, siRNA targeting zDHHC2, zDHHC3, and zDHHC15 elevated not only melanin but also tyrosinase protein levels [97]. zDHHC2 was found to co-localize with tyrosinase in the melanosomes of human epidermal melanocytes (NHEMs) [97]. Altogether, these modulators appear to be involved in regulating melanogenesis.

The molecular mechanism underlying the movement of melanosomes in melan-C (derived from albino mouse) melanocytes involves melanoregulin, whose limiting-membrane localization to this organelle is regulated by palmitoylation [105]. Specifically, melanoregulin contains six clustered cysteines at its N-terminus, which are needed for its membrane association to the melanosome [105].

### 3.7. Contribution of ErbB Family Members to Skin Health and Regulation by Palmitoylation

**Figure 6:**
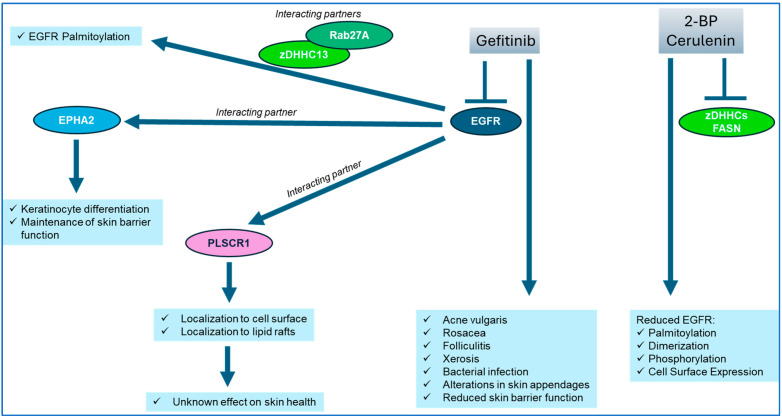
Contribution of EGFR to skin health and its regulation by palmitoylation. Targeting EGFR with gefitinib (TKI) leads to deterioration in skin health [151]. Targeting zDHHCs or FASN (with 2-BP or cerulenin, respectively) reduces EGFR palmitoylation, dimerization, and its cell surface expression [162]. EGFR palmitoylation can be regulated in a complex with Rab27A and zDHHC13 [168]. EGFR interacts with EPHA2, supporting skin barrier function [154], as well as PLSCR1, which is itself palmitoylated and is localized at the cell surface [170,171], although its role in skin health remains unclear.

*ErbB and Skin Health:* The ErbB family includes ErbB-1 (EGFR), ErbB-2 (c-neu), ErbB-3 (HER3), and ErbB-4 (HER4) [149]. While the activation of the EGFR pathway contributes to the elevated cellularity of skin tissues [150], EGFR-targeting agents such as gefitinib are well established to be associated with keratinocyte differentiation [150] and adverse responses in the skin, including loss of skin barrier function, leading to non-compliance by the patient [151]. These responses include acne vulgaris, rosacea, folliculitis, xerosis which may lead to bacterial infections, and changes in skin appendages such as hair abundance (i.e., hypertrichosis or alopecia) [151]. EGFR is critical for hair follicle development, and when disrupted, the resultant effect is the establishment of infections and the associated inflammatory response [152]. While EGFR has been the primary focal point within the ErbB family with respect to skin epidermal integrity, there are findings that link other ErbB family members to roles in the epidermis as well [153]. A summary is presented below in Figure 6.

EGFR associates with an array of interacting proteins including EPHA2, which contributes to keratinocyte function to support cellular differentiation via DSG1 as well as tight junction formation, implicating its role in the regulation of skin barrier function [154]. This was demonstrated using an EPHA2 knockdown strategy in epidermal keratinocytes, which increased the level of EGFR protein and the activation of MAPK [154]; on the other hand, the inhibition of EGFR activity (with AG-1478 inhibitor) recovered keratinocyte differentiation [154]. The phenotypic features associated with EGFR loss in postnatal murine keratinocytes are similar to those reported for ADAM17 loss [155]. ADAM17 is a metalloproteinase that is involved in the cleavage of EGFR ligands from the plasma membrane surface [155]. Along with defective skin barrier function, there was reduced transglutaminase activity and elevated keratin 1 and loricrin, as well as reduced involucrin and TGM3 [155]. The application of a proteomic approach to examine changes in protein levels in the epidermis from 3- and 10-day-old mice with a loss of either ADAM17 or EGFR identified reductions in components of the epidermal differentiation complex including involucrin, SPRP, corniferin-A, and keratin 1 and 10, as well as reduced filaggrin-2 and cathepsin E, along with increased kallikrein serine proteases (KLK10 and KLK6) [156].

Betacellulin (BTC) is one EGFR ligand whose transcript expression is reduced in atopic dermatitis skin lesions [157]. While BTC increased the expression of skin barrier function proteins (i.e., claudin-1, ZO1, loricrin, and filaggrin) in keratinocytes, an EGFR kinase inhibitor (i.e., AG-1478) opposed this response [157]. The expression of EGFR ligands themselves can be downregulated by the expression of gasdermin A (GSDMA), which is enriched in skin [158]; indeed, knockout models of GSDMA uncovered reduced RNA expression of EGFR ligands (i.e., amphiregulin, epiregulin, heparin-binding EGF-like growth factor, and epigen) [158]. Interestingly, the knockdown of filaggrin and loricrin using an siRNA-based approach reduced EGFR levels in HaCaT keratinocytes [159].

*Palmitoylation of ErbB1/EGFR:* Betacellulin, a ligand that interacts with ErbB family member receptors, is itself palmitoylated within its cytosolic region in its pro-form, which appears necessary for its stability and processing [160]. The dimerization of EGFR was demonstrated to require palmitoylation, induced by tyrosine kinase inhibitors (i.e., AEE788, gefitinib, and erlotinib) in a series of cancer cell lines including A549, MDA-MB-231, PC3, and DU145 [161]. Targeting FASN or zDHHCs with cerulenin or 2-BP, respectively, resulted in a diminished level of palmitoylated EGFR, dimerized EGFR, and phosphorylated EGFR in PC3 cells [162]. Further, mutagenesis of Cys797, Cys1025, and Cys1125 and their expression in HEK293 cells hindered EGFR dimerization [161]. Cell death induced by gefitinib was markedly elevated in MDA-MB-231 breast cancer cells, expressing a mutated EGFR (at Cys1025), or in cells treated with 2-BP [163].

While EGFR is predominantly localized to the cell surface, it can also be found in the mitochondria [164,165,166]. While the role of EGFR in the mitochondrial compartment remains unclear, the treatment of PC3 cancer cells with AEE788 (an EGFR inhibitor) causes mitochondrial fission while EGF-mediated activation of EGFR leads to fusion of the mitochondria [166]. It is further noted that EGFR palmitoylation was reduced by cerulenin (a FASN inhibitor) and AEE788 in PC3 cells [166]. Predicted palmitoylation sites (i.e., Cys781, Cys797, Cys1058, and Cys1146) were confirmed via mutagenesis and expression in HEK293T cells, which uncovered that Cys781 and Cys797 were palmitoylated sites on EGFR [166]. With FASN overexpression, the palmitoylation level of EGFR is elevated in PC3 cells [162]. Cerulenin or 2-BP treatment of PC3 cells caused increased EGFR localization to the lysosomal compartment (along with diminished plasma membrane localization) [162].

Endogenous levels of palmitoylated EGFR and its attainment of cell surface expression were reduced by 2-BP in MDA-MB-231 and A549 cells [167]. In the depalmitoylated state, EGFR was retained in the Golgi compartment [167]. The targeting of Golgi-localized DHHC enzymes identified that zDHHC13 and zDHHC17 altered the cell surface pattern of EGFR while DHHC13 altered the palmitoylation status of EGFR [167]. Specifically, Cys775, Cys797, Cys818, Cys939, Cys950, Cys1049, Cys1058, and Cys1146 were all found to acquire palmitoylation via mass spectrometric analyses and when these sites were collectively mutated (along with Cys781), EGFR lost its ability to be cell-surface localized [167]. With zDHHC13 knockdown, only Cys775, Cys781, and Cys797 were altered in their palmitoylation level [167]. Interestingly, EGFR and zDHHC13 co-immunoprecipitated in HEK293 cells [168]; moreover, Rab27A, found bound in a complex to zDHHC13, may alter the palmitoylated EGFR abundance (corresponding with low EGFR protein levels), as the knockdown of Rab27A reduced zDHHC13 mRNA levels [168]. In an independent report, when murine zDHHCs were overexpressed in A549 cells, EGFR activation and elevated palmitoylation were noted with zDHHC1, zDHHC2, and zDHHC21 [162]; these specific zDHHCs also co-immunoprecipitated with EGFR when co-expressed as tagged proteins in HEK293 cells [162]. The mutagenesis of Cys797 in EGFR followed by its overexpression in HEK293 cells reduced the palmitoylation state of EGFR as well its activation and dimerization ability [162]. Further, 2-BP treatment or a reduction in zDHHC20 expression (using shRNA) led to an increase in EGFR activation following EGF stimulation in MDA-MB-231 cells [169]. In contrast, overexpressed zDHHC20 led to elevated palmitoylated EGFR (and reduced activation) when expressed in HEK293T cells [169]. Deletion of the C-terminal tail containing palmitoylation sites caused the near-complete depletion of palmitoylated EGFR levels in these cells, and these sites were determined to be Cys1025 and Cys1034 via immunoprecipitation-ABE [169].

*Interaction of EGFR with Phospholipid Scramblase, PLSCR1:* Out of the numerous protein interactors of EGFR, one that is notable is phospholipid scramblase 1 (PLSCR1), which is itself palmitoylated [170,171]. PLSCR1 is an interferon-inducible (IFN) gene [134,172] that is localized to lipid rafts [170,173]; the loss of PLSCR1 using an shRNA-based approach led to a loss of cellular protection to the virulence factor, α-toxin, which is derived from *Staphylococcus aureus*, in A549 lung cancer cells [174]. A similar response was noted with 2-BP cellular treatment, which implicates palmitoylation in the protective response [174]. PLSCR1 contains a sequence rich in cysteine residues (CCCPCC) located at amino acid 184 to 189 [175]. Mutation of all the cysteines to alanines within this sequence resulted in PLSCR1 that was devoid of palmitoylation and its localization to the nuclear compartment in SVT2 murine fibroblasts [134]. Similarly, 2-BP caused PLSCR1 to be localized to the nuclear compartment in HT1080 cells [134]; some evidence supports the nuclear movement of PLSCR1 in an energy- and nuclear import receptor-dependent manner [176] along with a role as a transcriptional regulator of IP_3_R1 expression [177]. While the flavonoid wogonoside was demonstrated to depalmitoylate PLSCR1 and promote its nuclear movement in human acute myeloid leukemia U937 cells [178], the zDHHC involved remains to be determined. Other PLSCR family members that are reported to be palmitoylated include PLSCR2, which contains a similar palmitoylation motif to PLSCR1 but is localized to the nuclear compartment independent of its palmitoylation state [175,179]. With respect to PLSCR3, the cysteine residues that contribute to its palmitoylation status include Cys159, Cys161, Cys163, Cys164, and Cys166 [180]. Mutagenesis of these cysteines redirected PLSCR1 to the nuclear compartment from the mitochondria [180]. Further work is needed to investigate the role of PLSCR family members and their interaction with EGFR in skin health, which is currently limiting.

### 3.8. Expression of Skin Barrier Function Mediators, ErbB Family Members, and PLSCR Family Members in Skin Cancers

Using cBioportal [62,63,64], we assessed the expression of skin barrier function mediators, EGFR family members, and PLSCR family members across a series of skin cancers including basal cell carcinoma, cutaneous squamous cell carcinoma, acral melanoma, metastatic melanoma, and skin cutaneous melanoma. The method that was the most commonly applied was exome sequencing; other methods that were utilized included whole genome sequencing as well as targeting sequencing. Our analyses derived from cBioportal [62,63,64] are shown in Supplementary File S8 (skin barrier function proteins), Supplementary File S9 (ErbB family members), and Supplementary File S10 (PLSCR family members).

For skin barrier function proteins, across the claudins, few studies (up to two) showed an alteration frequency equal to or greater than 10%, for which the majority were mutations (followed by amplifications and deletions). In contrast, for desmocollin (DSC1, DSC2, and DSC3) and desmoglein (DSG1, DSG2, DSG3, and DSG4) genes, there were far more studies (up to 12 for DSG1) with an alteration frequency equal to or greater than 10%, which were mostly mutations. Similarly, for filaggrin (FLG) and filaggrin 2 (FLG2), there were up to 16 studies with an alteration frequency equal to or greater than 10% (the majority of which were mutations). For a detailed analysis of the relevant skin barrier function genes, please refer to Supplementary File S8. With respect to EGFR family members, we uncovered that there were four studies with an alteration frequency equal to or greater than 10% for EGFR, ErbB2, and ErbB3 while there were 15 studies with an alteration frequency equal to or greater than 10% (as high as 60%) for ErbB4. For all of these ErbB family members, the most common alteration was mutations followed by amplifications and deletions (along with a few structural variants and multiple alterations). For a detailed analysis, please refer to Supplementary File S9. For PLSCR family members, we uncovered that there were, in contrast, fewer studies (zero to one) with alteration frequencies equal to or greater than 10%. Similar to ErbB family members, the most frequent alterations were mutations followed by amplifications and deletions. For a detailed analysis, please refer to Supplementary File S10.

## 4. Targeting of S-Palmitoylation in Skin

### 4.1. Experimental Use of 2-Bromopalmitate and Limitations

While 2-bromopalmitate (2-BP), an analog of palmitic acid containing an a-bromine substitution, has been utilized to inhibit lipid metabolic processes in cells including fatty acid oxidation in mitochondria [181], monoacylglycerol acyltransferase [182], diacylglycerol acyltransferase [182], and fatty acid CoA ligase [183], it is commonly used as a general irreversible inhibitor of palmitoylation [184]. As noted below, high doses of 2-BP are required to hinder S-palmitoylation; regrettably, these doses are accompanied by cytotoxicity and cellular effects beyond the scope of S-palmitoylation (as discussed in [184]), including the inhibition of depalmitoylation enzymes (e.g., APT1 and APT2) [185] and other lipid modifications including N-myristoylation [186]. From a subset of articles focusing on specific search terms, an overview of the 2-BP doses utilized (μM range), cell lines, treatment times, and assessment outcomes is presented in Supplementary File S15 and discussed below.

*Use of 2-BP in Cell Lines:* A series of studies were reviewed that involved proteins defined to be palmitoylated and/or relevant to skin barrier function. Using a 3D skin model system, 25 μM 2-BP for a 17-day treatment period resulted in increased pigmentation of melanin, while up to 8 μM 2-BP treatment in NHEM and HM3KO cells increased not only cell number but also melanin content [97]. Specifically, 2-BP (50 μM, 48 h) treatment of HM3KO cells resulted in reduced palmitoylated levels of tyrosinase, which was accompanied by its increased stability [97]. Monkey kidney CV-1 fibroblasts overexpressing GFP-tagged melanoregulin were treated at 100 μM 2-BP for 8 h, after which lysosome staining was performed to demonstrate that the palmitoylation inhibitor reduced its localization to the lysosomes [105]. Melanoregulin, a palmitoylated protein, plays a role in the movement of melanosomes and their localization within melanocytes [105].

In A431 human epidermoid skin cancer cells, 50 μM 2-BP added to the growth media reduced the palmitoylated forms of plakophilin-2 and plakophilin-3 in the absence of alterations in the protein expression of desmosomal mediators [113]. In HEK293 cells overexpressing claudin-7 (another palmitoylated protein), 15 μM 2-BP altered its localization as determined by Western blotting analyses of sucrose density gradient fractions [115]. The protein levels of PADi3 (also known to be palmitoylated, expressed in HEK293T cells) were reduced following 100 and 200 μM 2-BP treatment for 36 h [124]. Reduced interactions between EpCAM, claudin-1, and claudin-4 to CD82 were noted following 50 μM 2-BP treatment for 20 h in ovarian cancer cell lines [187]. However, there are reports in which 2-BP treatment did not cause a change in either the expression or localization of palmitoylated proteins; this includes Cx32, which trafficked normally to gap junctions in LNCaP cells overexpressing Cx32 following 100 μM 2-BP treatment for 3 to 4.5 h [125]. The expression of protocadherin (PCDH7) in HeLa S3 cells that were treated with 100 μM 2-BP for an overnight period resulted in a change in its hydrophobicity [126]. With respect to b-catenin, another palmitoylated protein, 2-BP treatment in HEK293T cells (100 μM, 24 h) caused a reduction in its palmitoylation levels, along with reduced protein expression in HCT15 and RKO cells, which correlated with elevated ubiquitinated levels in HEK293T cells (100 μM, 6 h) [108]. δ-Catenin expression in M6A cells was also reduced along with diminished levels of palmitoylated levels [112]. Interaction between δ-catenin, a palmitoylated protein, and N-cadherin was hindered in hippocampal neurons under chemical-induced (glycine/bicuculline) long-term potentiation in combination with 50 μM 2-BP treatment (up to 40 min) [110]. The palmitoylated form of DSG2 (desmoglein-2) appears to be needed for the production of extracellular vesicles (EVs), per A431 treatment with 50 μM 2-BP for 18 h [136].

Other palmitoylated proteins were assessed with well-established roles in signaling in response to 2-BP. Treatment with 10 μM 2-BP for 2 h in WM3000 melanoma cells caused a reduction in the expression level of palmitoylated NRAS, while 20 μM 2-BP treatment for 3 min in HeLa S2 cells expressing EGFP-NRAS caused it to delocalize from the plasma membrane [188]. In CEM T lymphoblastic cells expressing a Cdc42 mutant (R186C), treatment with 2-BP at 30 μM caused the Cdc42 mutant to reduce its localization to the Golgi apparatus [106]. In B16F10 murine melanoma cells, 25 μM 2-BP treatment for either 30 min or 2 h prior to a 1 h heat shock resulted in the loss of Rac1 (a palmitoylated protein) cell surface localization in addition to reduced transcript levels of Hsp25 and Hsp70 [189]. In NPC neuronal progenitor cells, the localization of Drp1 (another palmitoylated protein) to the mitochondria was reduced following a 6-h treatment with 2-BP [123].

Other cell line studies not directly relevant to specific palmitoylated proteins include the following two reports. In human primary melanocytes and B16 melanoma cells, α-MSH treatment along with 2-BP (for which the doses and treatment times were undefined) diminished cAMP levels [121]. Furthermore, uveal melanoma cells expressing GNAQ/11 and treated with up to 35 μM 2-BP for 72 h, reduced cellular proliferation while up to 100 μM 2-BP reduced the phosphorylation level of ERK1/2 along with increasing the cleaved PARP [190].

*Use of 2-BP in Rodent Models:* While oxaliplatin induced an increase in δ-catenin in rats, 2-BP treatment led to a reduction in palmitoylated δ-catenin levels [111]. These molecular-level changes were accompanied by a reduction in mechanical allodynia, as assessed via paw withdrawal studies [111]. Nanoparticles composed of 2-BP and camptothecin (PLNs, polymer-lipid nanoparticles) were developed to target PD-L1 as a novel immune checkpoint blockade therapy [191]; it was tested in vivo using C57BL/6 mice that were implanted with the tumorigenic B16-F10 cell line (40 mg/kg 2-BP) [191]. The PLN inhibited the progression of the tumor in vivo and extended the lifespan of the mice [191].

### 4.2. Other Compounds and Strategies Targeting S-Palmitoylation

**Figure 7:**
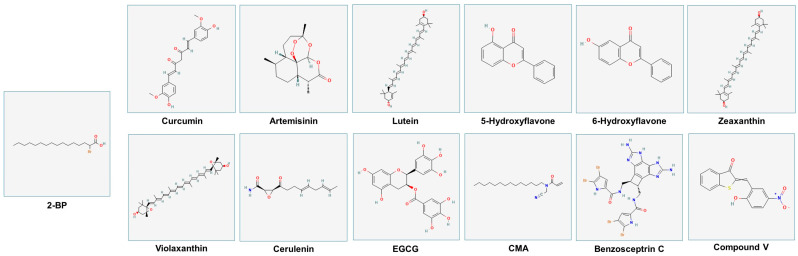
Structures of palmitoylation inhibitors. Chemical structures were obtained from PubChem [196]. The PubChem ID# for 2-bromopalmitate (2-BP) is #82145. Further information about the remaining compounds, including molecular weight, PubChem ID#, and molecular formula, can be found in Supplementary File S16.

Figure 7 presents the structures of a subset of compounds (as derived from PubChem [192]), for which there is evidence of their ability to inhibit S-palmitoylation.

Due to the active site conservation across zDHHCs, the development of specific zDHHC inhibitors remains a challenge. However, the variation in the N- and C-termini of zDHHCs may support the development of strategies to hinder the recruitment of their substrates, which could be more promising.

*Curcumin:* Derived from *Curcuma longa*, curcumin (structurally composed of two aromatic rings that are linked by unsaturated carbonyl groups) has been shown to target integrin β4, which is palmitoylated within its cytosolic segment by DHHC3 [193]; this lipid modification may be essential for the lipid raft localization of integrin β4, to support signaling events [193]. When curcumin was utilized at 15 μM in breast cancer cell lines (i.e., MDA-MB-231 and MDA-MB-435), the palmitoylation of integrin β4 was reduced [193]. In HCC1806 cells, other targets of curcumin were identified, including flotillin-2, DHHC5, and syntaxin-6 [193].

*Artemisinin (ART):* ART, which contains an endoperoxide group, alters the palmitoylation state of the transferrin receptor [194,195]. Due to its restricted permeability into cells, a derivative called ART-yne was generated with improved cellular permeability [196]. Breast cancer MCF-7 cells treated with 30 μM ART or ART-yne (24 h) identified zDHHC6 as a co-interacting partner [196]; as a result, the palmitoylation status of zDHHC6 substrates was altered including H-Ras [196].

*Plant-Derived Natural Carotenoid and Flavonoids:* Towards the goal of identifying palmitoylation inhibitors that elicit reduced toxicity and increased specificity, 115 natural compounds were investigated using the zDHHC20 binding pocket and Cys156, which is essential in the nucleophilic attack on the acyl-CoA donor [197]. The in silico screening identified 24 top hits including lutein, 5-hydroxyflavone, and 6-hydroxyflavone [197]. These three agents elicited an improved zDHHC20 groove blocking capacity and binding affinities with the most stable conformation induced with lutein [197]. In an independent report, lutein (0.15 μM) and other xanthophylls (i.e., zeaxanthin (0.45 μM) and violaxanthin (0.6 μM)) were assessed using HEK293F cells for their ability to inhibit the palmitoylation status of β-carotene oxygenase 2 (BCO2) [197]. Altogether, further research is necessary to determine the effectiveness of these natural agents experimentally.

*Tunicamycin and Cerulenin:* These inhibitors, like 2-BP, are lipid-based compounds [198]. Tunicamycin (2.5 μM, 48 h) and cerulenin (25 μM, 48 h) induce stress in the endoplasmic reticulum, leading to apoptotic features in cells, via G2 arrest in glioblastoma cells [198]. It is proposed that XBP1 palmitoylation diminishes with treatment, leading to these functional responses [198]. While the mechanism of action for tunicamycin remains unclear, it has been proposed that it may elicit activity as a competitive inhibitor of palmitoyl-CoA [198]. In contrast, cerulenin may bind through its epoxy carboxamide moiety to the catalytic cysteine of the DHHC enzyme [198].

*Derivative of (−)-Epigallocatechin-3-Gallate (EGCG):* Derived from green tea, the EGCG polyphenol and derivatives (50 mM, 6 h) containing variable hydrocarbon tails were investigated for their ability to alter global palmitoylation profiles in HeLa cells [199]. In contrast to short-tailed hydrocarbon chains, the long-tailed chains (14 and 16) were effective in mediating alterations in palmitoylation; further, EGCG itself was without effect [199]. From a mechanistic perspective, in silico analyses uncovered that EGCG derivatives fit into the hydrophobic groove in DHHC20, similar to 2-BP [199]. Specifically, W158, F171, F174, and L227 of DHHC20 interacted with the acyl chain, whereas the catalytic site H154 mediated interactions with the cysteine residue to support the interaction between the active site of DHHC20 and the hydrocarbon chain of the derivative [199].

*Cyano-myracrylamide (CMA):* Along with reduced cytotoxicity (up to 40 μM) and improved specificity, CMA (containing an “acrylamide warhead” along with a cyanomethyl group and a 14-carbon hydrocarbon tail) elicits increased potency against zDHHC20 (IC_50_ of 1.35 μM) and can thus be utilized at lower doses compared to 2-BP [200]. CMA, in contrast to 2-BP, elicited little to no effect on APT1 and APT2 [200]. Global S-palmitoylation was also reduced in CMA-treated HEK293T cells [200]; one target from this study that was examined in more depth included EGFR, whose palmitoylation status was reduced in MDA-MB-231 cells [200].

*Benzosceptrin C:* From a drug panel screen of 300 Chinese traditional medicines using the RKO colorectal cancer cell line, benzosceptrin C (10 μM, 24 h) was identified to inhibit the activity of zDHHC3 which hindered the palmitoylation of a downstream target, namely programmed cell death ligand-1 (PD-L1) [201]. Benzosceptrin C interacted directly with DHHC via co-immunoprecipitation, and the binding analyses identified hydrogen bonds between this marine natural product to the Thr176, Cys156, and Glu223 residues of DHHC3 [201]. Furthermore, benzosceptrin C induced T-cell cytotoxicity to colorectal cancer cells [201].

*2-(2-Hydroxy-5-nitro-benzylidene)-benzo[b]thiophen-3-one (Compound V):* In comparison to 2-BP, although compound V was able to inhibit zDHHC enzymes, it was less potent, induced broader effects on targets (10–100 μM), and was accompanied by solubility issues [202]. Compound V inhibited the autoacylation property of DHHC enzymes similarly to 2-BP; however, 2-BP had increased potency [202]. While 2-BP is an irreversible inhibitor, compound V was determined to be reversible [202].

*Nitrofuran Derivatives:* From a screen to identify inhibitors of STING (a signaling molecule involved in type I interferon production, relevant to the pathogenesis of skin cancers, and previously targeted by 2-BP at 50 μM [203]), C-176 and C-178 were shown to hinder the palmitoylation state of murine STING without altering other targets, including the transferrin receptor or calnexin [204]. Through SAR analysis, the furan and nitro group of these compounds appear necessary to elicit their inhibitory activities [204]; further, it is proposed that a covalent linkage may form between Cys91 of STING and C-178 [204]. Compounds that could also target the human form of STING included C-170 and C-171, in a mechanism similar to that described for the murine form [204]. A second screen identified that H-151 (0.02–2 μM) potently inhibited the human STING, including its palmitoylation status which occurred in an irreversible manner dependent on Cys91 [204].

*Proteolysis-targeting chimaeras (PROTACs):* PROTACs are composed of a ligand specific to a protein target (e.g., zDHHCs), a linker region, and another ligand that houses the components involved in ubiquitination (e.g., E3 ubiquitin ligase) [205]. This is proposed as a strategy to target proteins that are considered “undruggable” [205]. A recent study demonstrated the feasibility of PROTAC targeting of Halo-tagged zDHHC5 and zDHHC20 using FT-293 cells, which uncovered reduced palmitoylation of their specific substrates that were assessed [206].

## 5. Limitations in Research Area, Limitations in the Study, and Future Considerations

**Figure 8:**
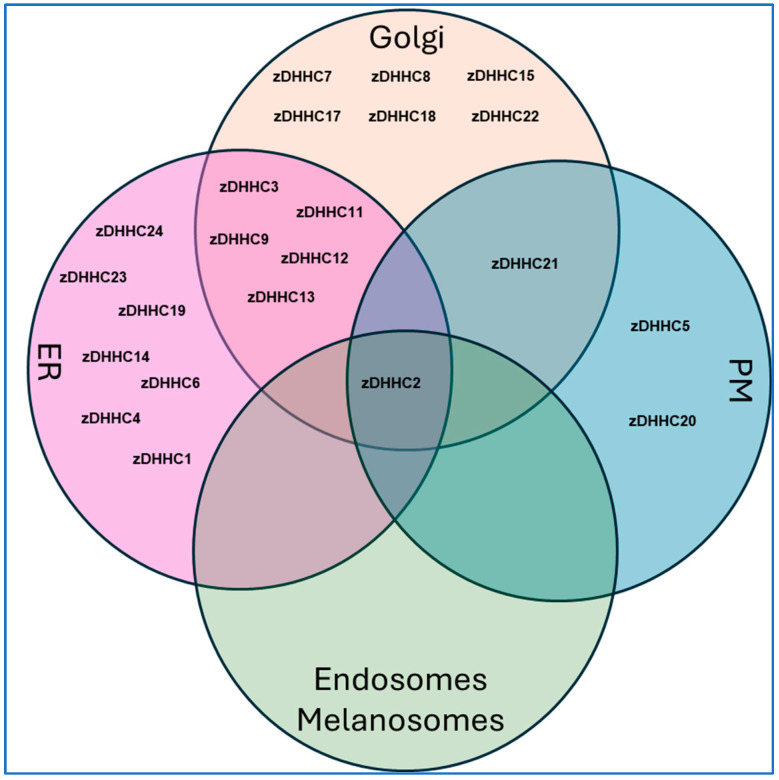
Localization of overexpressed tagged zDHHCs in mammalian cell lines. The Venn diagram organizes the experimentally determined localization patterns of zDHHCs. Please refer to Supplementary File S17.

Using the accession number for article [207], we have validated the DHHC nomenclature for the appropriate human zDHHC annotation. As shown in Figure 8 and Supplementary File S17, we have summarized the localization for each zDHHC reported in mammalian cells. As noted, the cell lines utilized to determine their intracellular localization include HEK293T/HEK293, COS7, HeLa, and PC12, with only a few studies reported using cell lines relevant to skin health such as Normal Human Epidermal Melanocytes (NHEMs). All of the studies reported in Figure 8 (presented below) are derived from overexpression studies using tagged DHHCs followed by visualization via immunofluorescence using colocalization markers for the endoplasmic reticulum, the stacks of the Golgi apparatus, endosomes, or melanosomes. As an overview, the zDHHCs appear to be predominantly localized to the endoplasmic reticulum as well as Golgi stacks, with a smaller subset of zDHHCs located to the endosomes and the plasma membrane. The zDHHC localization appears to vary across different cell types and/or independent studies. While overexpressed zDHHC1 [207], zDHHC6 [23,25,207], zDHHC14 [207], zDHHC19 [207], zDHHC23 [207], and zDHHC24 [207] were localized to the ER and zDHHC7 [207,208,209], zDHHC8 [207,208], zDHHC15 [97,207,209], zDHHC17 [207,209], zDHHC18 [207], and zDHHC22 [209] were localized to the Golgi stacks, the localization of zDHHC2 differed across varying cell lines from the ER [207] to the Golgi [208], endosomes [210], and PM [210,211], and to melanosomes in NHEM cells [97]. Furthermore, other zDHHCs with varying localization patterns included zDHHC3 [25,97,207,208,209], zDHHC4 [25,207], zDHHC9 [207,209], zDHHC11 [207,209], zDHHC12 [207], zDHHC13 [207,209], and zDHHC21 [103,207,208,209]. In contrast, two zDHHCs were reported to be localized primarily at the PM including zDHHC5 [207] and zDHHC20 [207]. Per our literature search terms, zDHHC16 was not determined due to its low expression level [207].

As noted in Supplementary File S17, ER retention signals were uncovered (e.g., KKXX motifs) which appears to be responsible for the retention of specific zDHHCs to the endoplasmic reticulum. Unfortunately, endogenous localization data appear to be lacking, possibly due to a limitation in antibody sensitivity capable of detecting endogenous zDHHCs for immunofluorescence studies. Advancements in experimental tools for endogenous zDHHC detection in cell lines relevant to skin health would support advancements in this research area.

Although the Human Protein Atlas [55,56,57,58,59,60,61] uncovered RNA expression levels for a subset of zDHHCs across skin-relevant cell types (e.g., basal keratinocytes, basal squamous epithelial cells, melanocytes, squamous epithelial cells, and suprabasal keratinocytes), there are currently limited data available at the protein level across these specific cell types, likely for the reasons described above with regards to antibody limitations. In addition, another research area that requires further work includes RNA or protein expression profiling for zDHHCs as well as the above-described skin barrier function mediators across specific skin disease tissues and skin-specific cell types.

Since our study exclusively utilized PubMed, this could have led to the exclusion of relevant articles available from other sources. While our literature searches were extensive across an array of terms, this may have excluded potentially relevant articles with respect to skin. Since we focused primarily on 13 diseases of the skin, there may be palmitoylation studies for other diseases relevant to skin that we did not investigate in our studies. Altogether, S-palmitoylation appears to influence several cellular processes necessary for maintaining skin homeostasis. While 2-bromopalmitate (2-BP) has limited potential pharmaceutical applications due it its insolubility in water and lack of substrate specificity, experiments integrating 2-BP into nanoparticles have shown promise. However, further research efforts into developing novel inhibitors are direly needed to create selective and effective pharmaceuticals targeting S-palmitoylation, specifically those for unique zDHHCs. Moreover, we propose that studies could be designed to investigate the palmitoylation-dependent interactions between EGFR and PLSCR1 within lipid raft regions. Altogether, we propose that manipulation of the S-palmitoylation of skin barrier proteins specifically in skin tissues may provide an innovative approach to combating skin diseases such as atopic dermatitis and psoriasis.

## Figures and Tables

**Table 1:** Proximity of human zDHHCs to GWAS locus sites in atopic dermatitis and psoriasis, as well as their predicted palmitoylation sites. The table lists the UniProt ID#, HGNC ID#, and chromosomal location for the human zDHHCs. SwissPalm was used to derive their palmitoylation sites as well as any validated experiments reported. The GWAS locus sites are listed for susceptibility risk with respect to atopic dermatitis and psoriasis.

Human DHHC	UniProt ID#	HGNC ID#	HGNC Chromosomal Location	Located Within or in Proximity to an Atopic Dermatitis GWAS Locus	Located Within or in Proximity to a Psoriasis GWAS Locus	SWISSPALM	
						Validated Experimentally	Predicted Palmitoylation Sites
zDHHC1	Q8WTX9	17916	16q22.1			0 experiments	Cys7, Cys397
zDHHC2	Q9UIJ5	18469	8p22			0 experiments	Cys14
zDHHC3	Q9NYG2	18470	3p21.31			0 experiments	Cys132, Cys133 (isoform 1); Cys132, Cys133 (isoform 2)
zDHHC4	Q9NPG8	18471	7p22.1	7p22.2		1 experiment	Cys22, Cys24
zDHHC5	Q9C0B5	18472	11q12.1			2 experiments	Cys236, Cys237 (isoform 1); Cys183, Cys184 (isoform 2)
zDHHC6	Q9H6R6	19160	10q25.2			8 experiments	Cys5, Cys20, Cys101, Cys135, Cys138, Cys328 (isoform 1); Cys5, Cys20, Cys131, Cys132, Cys324 (isoform 2)
zDHHC7	Q9NXF8	18459	16q24.1			1 experiment	Cys132, Cys135, Cys136, Cys137 (isoform 1); Cys169, Cys172, Cys173, Cys174 (isoform 2)
zDHHC8	Q9ULC8	18474	22q11.21		22q11.21	1 experiment	Cys236 (isoform 1); Cys144, Cys145 (isoform 2); Cys236 (isoform 3)
zDHHC9	Q9Y397	18475	Xq26.1			1 experiment	Cys24, Cys25, Cys158. Cys283
zDHHC11	Q9H8X9	19158	5p15.33			1 experiment	Cys144
zDHHC12	Q96GR4	19159	9q34.11	9q34.4		0 experiments	
zDHHC13	Q8IUH4	18413	11p15.1	11p15.4	11p15.4	1 experiment	Cys10, Cys447 (isoform 1); Cys271 (isoform 2); Cys317 (isoform 3)
zDHHC14	Q8IZN3	20341	6q25.3		6q25.3	0 experiments	Cys12, Cys311, Cys357 (isoform 1); Cys12, Cys311 (isoform 2)
zDHHC15	Q96MV8	20342	Xq13.3			0 experiments	Cys15, Cys16 (isoform 1); Cys15, Cys16 (isoform 2); Cys15, Cys16 (isoform 3)
zDHHC16	Q969W1	20714	10q24.1		10q24.31	0 experiments	
zDHHC17	Q8IUH5	18412	12q21.2			0 experiments	Cys551, Cys602 (isoform 1); Cys10 (isoform 2)
zDHHC18	Q9NUE0	20712	1p36.11	1p36.11	1p36.11	1 experiment	Cys4, Cys339 (isoform 1); Cys3, Cys21, Cys204 (isoform 2)
zDHHC19	Q8WVZ1	20713	3q29			1 experiment	Cys114, Cys117, Cys165 (isoform 1); Cys114, Cys117, Cys165 (isoform 2)
zDHHC20	Q5W0Z9	20749	13q12.11		13q12.11	0 experiments	Cys9, Cys10 (isoform 1); Cys9, Cys10 (isoform 2); Cys9, Cys10 (isoform 3); Cys9, Cys10 (isoform 4)
zDHHC21	Q8IVQ6	20750	9p22.3			0 experiments	Cys15, Cys16, Cys218, Cys219
zDHHC22	Q8N966	20106	14q24.3			0 experiments	Cys17, Cys203, Cys204
zDHHC23	Q8IYP9	28654	3q13.31	3q13.2		0 experiments	Cys24, Cys25, Cys26
zDHHC24	Q6UX98	27387	11q13.2	11q13.1	11q13.1	0 experiments	

**Table 2:** Proximity of human skin barrier function proteins to GWAS locus sites in atopic dermatitis and psoriasis, as well as their predicted palmitoylation sites. The table lists the UniProt ID#, HGNC ID#, and chromosomal location of a subset of skin barrier function proteins. SwissPalm was used to derive their palmitoylation sites as well as any validated experiments reported. The GWAS locus sites are listed for susceptibility risk with respect to atopic dermatitis and psoriasis.

Skin Barrier Function Protein	UniProt ID#	HGNC ID#	Chromosomal Location	Located Within or in Proximity to an Atopic Dermatitis GWAS Locus	Located Within or in Proximity to a Psoriasis GWAS Locus	SWISSPALM	
						Validated Experimentally	Predicted Palmitoylation Sites
Claudin-1	O95832	2032	3q28			0 experiments	Cys107, Cys183, Cys184, Cys186
Claudin-2	P57739	2041	Xq22.3			0 experiments	
Claudin-3	O15551	2045	7q11.23			3 experiments	Cys23, Cys24, Cys181, Cys182, Cys184
Claudin-4	O14493	2046	7q11.23			1 experiment	Cys24, Cys25, Cys182, Cys183, Cys185
Claudin-5	O00501	2047	22q11.21		22q11.21	0 experiments	Cys14, Cys182, Cys183
Claudin-6	P56747	2048	16p13.3	16p13.13	16p13.13	1 experiment	Cys182, Cys183, Cys185
Claudin-7	O95471	2049	17p13.1			0 experiments	Cys24, Cys107 (isoform 1); Cys24, Cys107 (isoform 2)
Claudin-14	O95500	2035	21q22.13		21q22.11	2 experiments	Cys185
Occludin	Q16625	8104	5q13.2			0 experiments	
ZO1	Q07157	11827	15q13.1		15q13.3	0 experiments	Cys1718 (isoform 1); Cys1638 (isoform 2)
Connexin26	P29033	4284	13q12.11		13q12.11	0 experiments	Cys169 (predicted but not validated)
Connexin32	P08034	4283	Xq13.1			0 experiments	Cys280
Afadin	P55196	7137	6q27			0 experiments	
Nectin-1a	Q15223	9706	11q23.3	11q23.3		0 experiments	
Nectin-2a	Q92692	9707	19q13.32	19q13.2	19q13.33	0 experiments	
Nectin-3a	Q9NQS3	17664	3q13.13	3q13.2	3q13	0 experiments	Cys11 (isoform 1); Cys11 (isoform 2); Cys8 (isoform 3)
Integrin a6b4	P23229	6142	2q31.1			5 experiments	Cys8, Cys1078 (isoform 1); Cys8, Cys1039 (isoform 2); Cys8, Cys1039 (isoform 3); Cys8, Cys1034 (isoform 4); Cys8, Cys1034 (isoform 5); Cys8, Cys1078 (isoform 6); Cys17, Cys920 (predicted but not validated) (isoform 7); Cys8, Cys1063 (isoform 9)
b-Catenin	P35222	2514	3p22.1			4 experiments	Cys8 (isoform 2)
d-catenin	O60716	2515	11q12.1			2 experiments	
Plakophilin-1	Q13835	9023	1q32.1		1q32.1	0 experiments	Cys14, Cys206, Cys692 (isoform 1); Cys14, Cys206, Cys671 (isoform 2)
Plakophilin-2	Q99959	9024	12p11.21			1 experiment	
Plakophilin-3	Q9Y446	9025	11p15.5	11p15.4	11p15.4	2 experiments	
Plakophilin-4	Q99569	9026	2q24.1	2q24.3	2q24.2	0 experiments	Cys939, Cys940 (isoform 1 and 2)
Plakoglobin	P14923	6207	17q21.2	17q21.2	17q21.2	1 experiment	
Periplakin	O60437	9273	16p13.3	16p13.13	16p13.13	0 experiments	
Plectin	Q15149	9069	8q24.3	8q24.14		0 experiments	Cys848, Cys849, Cys850, Cys3110 (isoform 1); Cys738, Cys739, Cys740, Cys3000 (isoform 2); Cys734, Cys735, Cys736, Cys2996 (isoform 3); Cys711, Cys712, Cys713, Cys2973 (isoform 4); Cys711, Cys712, Cys713, Cys2973 (isoform 5); Cys20, Cys39, Cys40, Cys715, Cys716, Cys717, Cys2977 (isoform 6); Cys679, Cys680, Cys681, Cys2941 (isoform 7); Cys689, Cys690, Cys691, Cys2951 (isoform 8); Cys697, Cys698, Cys699, Cys2959 (isoform 9)
Desmoglein-1	Q02413	3048	18q12.1	18q12.1		0 experiments	Cys570, Cys571, Cys573, Cys735 (isoform 1); Cys94 (isoform 2)
Desmoglein-2	Q14126	3049	18q12.1	18q12.1		3 experiments	Cys17, Cys635, Cys637, Cys813
Desmoglein-3	P32926	3050	18q12.1	18q12.1		1 experiment	Cys641, Cys833, Cys834
Desmoglein-4	Q86SJ6	21307	18q12.1	18q12.1		0 experiments	Cys653, Cys654, Cys655, Cys820 (isoform 1); Cys653, Cys654, Cys655, Cys839 (isoform 2)
Desmocollin-1	Q08554	3035	18q12.1	18q12.1		0 experiments	Cys713, Cys864 (isoform 1); Cys711 (isoform 2)
Desmocollin-2	Q02487	3036	18q12.1	18q12.1		1 experiment	Cys716, Cys871 (isoform 1); Cys716 (isoform 2)
Desmocollin-3	Q14574	3037	18q12.1	18q12.1		0 experiments	Cys712, Cys866
Envoplakin	Q92817	3503	17q25.1		17q25.3	0 experiments	Cys324
Corneodesmosin	Q15517	1802	6p21.33	6p21.33		0 experiments	
Filaggrin	P20930	3748	1q21.3	1q21.3	1q21.3	0 experiments	
Filaggrin-2	Q5D862	33276	1q21.3	1q21.3	1q21.3	0 experiments	Cys379
Keratinocyte proline-rich protein	Q5T749	31823	1q21.3	1q21.3	1q21.3	0 experiments	Cys2, Cys9, Cys16, Cys17, Cys333, Cys334, Cys399
Cornifelin	Q9BYD5	30183	19q13.2		19q13.33	0 experiments	Cys31, Cys59, Cys60, Cys61
Small proline-rich protein 2D	P22532	11264	1q21.3	1q21.3	1q21.3	0 experiments	Cys8, Cys18, Cys23, Cys27, Cys36, Cys41, Cys45
Small proline-rich protein 2E	P22531	11265	1q21.3	1q21.3	1q21.3	0 experiments	Cys8, Cys18, Cys23, Cys27, Cys36, Cys41, Cys45
Involucrin	P07476	6187	1q21.3	1q21.3	1q21.3	0 experiments	
Loricrin	P23490	6663	1q21.3	1q21.3	1q21.3	0 experiments	
PADi3	Q9ULW8	18337	1p36.13	1p36.11	1p36.11	0 experiments	Cys610, Cys612
Transglutaminase 1	P22735	11777	14q12			1 experiment	Cys47, Cys50, Cys51, Cys53, Cys471, Cys472 (isoform 1); Cys29, Cys30 (isoform 2)
Transglutaminase 2	P21980	11778	20q11.23			0 experiments	Cys10, Cys230, Cys370, Cys371 (isoform 1); Cys10, Cys230, Cys370, Cys371 (isoform 2); Cys10, Cys230 (isoform 3)

**Table 3:** Methodologies applied to assay palmitoylation. The table summarizes the respective methods used to assay palmitoylation with respect to the PMID listed. The most common methods utilized include acyl-biotin exchange (ABE), metabolic labeling, and click chemistry. The PMIDs are organized in chronological order from 1996 to 2024. Refer to Supplementary File S12 for additional details.

PMID #	Publication Year	Palmitoylation Method
8824274	1996	Metabolic labeling using [1-^14^C]-palmitic acid
17762858	2008	
		
8824274	1996	Metabolic labeling using [^3^H]-palmitic acid
9415709	1997	
15769849	2005	
17574235	2007	
19956733	2009	
22314500	2012	
22940130	2012	
32283203	2020	
		
20548961	2010	Acyl-biotin exchange (ABE) assay
24562000	2014	
25002405	2014	
25944911	2015	
26054340	2015	
26121212	2015	
26288350	2015	
26334723	2015	
27120791	2016	
27703000	2016	
28869973	2017	
29038583	2017	
29588412	2018	
31402609	2019	
31669413	2019	
34146657	2021	
36701140	2023	
36762613	2023	
36878899	2023	
37865665	2023	
38697304	2024	
		
28526873	2017	Alkylating resin-assisted capture (RAC) assay
31669413	2019	
36063887	2022	
		
25002405	2014	Metabolic labeling using 17-ODYA
36063887	2022	
		
25944911	2015	Click chemistry
28008916	2016	
32963941	2020	
37865665	2023	
37865665	2023	
		
16645047	2006	Mass spectrometry
26121212	2015	
29097667	2017	
		
21617949	2012	Prediction using CSS-Palm 2.0, 3.0, or 4.0
25120100	2014	
28932213	2017	
36063887	2022	

**Table 4:** Evidence-based determination of palmitoylation status for a subset of skin barrier function proteins. The table lists the different skin barrier proteins that have been experimentally determined to be palmitoylated.

Skin Barrier Function Protein	Species	Experimentally Assessed Palmitoylation Status
β-Catenin	Human	Detected
Catenin-δ-1	Mouse	
δ-Catenin	Rat, Mouse, Human	
Claudin-1	Canine	
Claudin-2	Human, Canine	
Claudin-3	Human	
Claudin-4	Human	
Claudin-6	Human	
Claudin-7	Human, Rat	
Claudin-14	Human	
Connexin32	Mouse, Rat	
Corniferin-A	Mouse	
Corniferin-B	Mouse	
Desmocollin-2	Human	
Desmoglein-3	Human, Mouse	
EGFR	Human	
Filaggrin-2	Mouse	
Integrin a6b4	Human	
Involucrin	Mouse	
Keratinocyte proline-rich protein	Mouse	
Loricrin	Mouse	
PADi3	Mouse	
Plakoglobin	Human	
Plakophilin-2	Human	
Plakophilin-3	Human	
Small proline-rich protein 2D, 2I, 2E	Mouse	
Transglutaminase 1	Human, Mouse	
Transglutaminase A	Drosophila	
		
Connexin26	Mouse	Not Detected
Occludin	Canine	
Transglutaminase B	Drosophila	

**Table 5:** SwissPalm predicted palmitoylation sites for the ErbB and PLSCR families. The human forms of ErbB and scramblase members listed with respect to their UniProt ID#, HGNC ID#, chromosomal location, number of validated experiments, and predicted palmitoylation sites.

ErbB Family					
ErbB Family	UniProt ID#	HGNC ID#	Chromosomal Location	SWISSPALM	
				Validated Experimentally	Predicted Palmitoylation Sites
ErbB1	P00533	3236	7p11.2	5 experiments	Cys19 (isoform 1); Cys19 (isoform 2); Cys19 (isoform 3); Cys19 (isoform 4)
ErbB2	P04626	3430	17q12	1 experiment	Cys7 (isoform 1); Cys16 (isoform 2); Cys611 (isoform 4); Cys23, Cys205 (isoform 5); Cys7, Cys771 (isoform 6)
ErbB3	P21860	3431	12q13.2	0 experiment	Cys167, Cys168, Cys264, Cys268 (isoform 4)
ErbB4	Q15303	3432	2q34	0 experiment	Cys1106 (isoform 1); Cys1096 (isoform 2); Cys1090 (isoform 3); Cys1080 (isoform 4)
**PLS Family**					
PLSCR1	O15162	9092	3q24	5 experiments	Cys148, Cys153, Cys154, Cys181, Cys184, Cys185, Cys186, Cys188, Cys189, Cys239, Cys240 (isoform 1); Cys67, Cys72, Cys73, Cys100, Cys103, Cys104, Cys105, Cys107, Cys108, Cys158, Cys159 (isoform 2)
PLSCR2	Q9NRY7	16494	3q24	0 experiment	Cys10, Cys68, Cys69, Cys96, Cys98, Cys99, Cys100, Cys101, Cys103, Cys104 (isoform 1); Cys9, Cys141, Cys142, Cys169, Cys171, Cys172, Cys173, Cys174, Cys176, Cys177 (isoform 2); Cys137, Cys138, Cys165, Cys167, Cys168, Cys169, Cys170, Cys172, Cys173 (isoform 3)
PLSCR3	Q9NRY6	16495	17p13.1	0 experiment	Cys125, Cys130, Cys158, Cys162, Cys163, Cys165
PLSCR4	Q9NRQ2	16497	3q24	0 experiment	Cys136, Cys197, Cys198, Cys199, Cys201, Cys202
PLSCR5	A0PG75	19952	3q24	0 experiment	
ANO6	Q4KMQ2	25240	12q12	0 experiment	Cys3 (isoform 3); Cys869, Cys871 (isoform 4)
XKR8	Q9H6D3	25508	1p35.3	0 experiment	Cys345, Cys346

## Data Availability

All the data are presented within the manuscript text and associated files.

## Supplementary Materials

The following supporting information can be downloaded at https://www.mdpi.com/article/10.3390/ijms26041673/s1.

## Abbreviations

2-BP, 2-bromopalmitate; 3D, 3-dimensional; 17-ODYA, 17-octadecynoic acid; ABE, acyl-biotin exchange; AJ, adherens junction; ADAM17, ADAM metalloproteinase domain 17;AKT, protein kinase B; a-MSH, a -melanocyte stimulating hormone; AMPK, AMP-activated protein kinase; APT, acyl thioesterase; ART, artemisinin; BRAF, B-Raf Proto-Oncogene, serine/threonine kinase; BTC, betacellulin; cAMP, cyclic AMP; Cas9, CRISPR associated protein 9; CDC42, cell division cycle 42; CMA, cyano-myracrylamide; CRISPR, clustered regularly interspaced short palindromic repeats; CSS, clustering and scoring strategy; Cx26, connexin 26; Cx32, connexin 32; DHHC, Asp-His-His-Cys tetrapeptide motif; Drp1, dynamin-related protein 1; DSC, desmocollin; DSG, desmoglein; EGCG, epigallocatechin-3-gallate; EGFR/ErbB1, epidermal growth factor receptor; eNOS, endothelial nitric oxide synthase; ENU, N-ethyl-N-nitrosourea; ErbB2, Erb-B2 receptor tyrosine kinase; ErbB3, Erb-B3 receptor tyrosine kinase; ErbB4, Erb-B4 receptor tyrosine kinase; EpCAM, epithelial cell adhesion molecule; EphA2, Ephrin type-A receptor 2; ER, endoplasmic reticulum; FASN, fatty acid synthase; FLG, filaggrin; FLG2, filaggrin 2; GEPIA, Gene Expression Profiling Interactive Analysis; GPCR, G-protein coupled receptor; GSDMA, gasdermin A; GWAS, genome-wide association studies; HGNC, HUGO Gene Nomenclature; HM3KO, human melanoma cells; HPA, Human Protein Atlas; H-Ras, HRas proto-oncogene GTPase; IFN, interferon; IL, interleukin; IP, immunoprecipitation; IPA, Ingenuity Pathway Analyses; JAK, Janus kinase; kDa, kilodalton; KLK, kallikrein; Lck, T-cell-specific protein tyrosine kinase; MALDI-TOF-MS, Matrix-Assisted Laser Desorption/Ionization Time-of-Flight Mass Spectrometry; MAPK, mitogen-activated protein kinase; MC1R, melanocortin 1 receptor; MCAM, melanoma cell adhesion molecule; MITF, melanocyte inducing transcription factor; mRNA, messenger ribonucleic acid; MS, mass spectrometry; NHEK, normal human epidermal keratinocytes; NHEM, human epidermal melanocytes; PADi3, peptidyl arginine deiminase 3; PARP, poly(ADP-ribose) polymerase; PAT, palmitoyl acyltransferase; PCDH7, protocadherin 7; PD-L1, programmed death-ligand 1; PDZ, post-synaptic density-95, disks-large, and zonula occludens-1; PI3K, phosphatidylinositol-4,5-bisphosphate-3-kinase; PLN, polymer-lipid nanoparticles; PLSCR, phospholipid scramblase; PTM, post-translational modification; RAC, resin-assisted capture; RHC, red hair color; RNA, ribonucleic acid; SH3, SRC-homology 3 domain; SPRP, small protein-rich proteins; STAT, signal transducer and activator of transcription; STING, stimulator of interferon response cGAMP interactor 1; TCGA, The Cancer Genome Atlas; TGM1, transglutaminase 1; TJ, tight junction; TM, transmembrane; TMEM16F, transmembrane protein 16F; TNF, tumor necrosis factor; TPA, 12-O-tetracecanoylphorbol-13-acetate; TRYP, tyrosinase-related protein; UniProt, Universal Protein Resource; UVR, ultraviolet radiation; XBP1, X-Box binding protein 1; ZO-1, zonula occludens-1; XKR8, XK-related 8; zDHHC, zinc finger DHHC-type containing.
