# Supplementary material for: zDHHC-Mediated S-Palmitoylation in Skin Health and Its Targeting as a Treatment Perspective
Source: Int J Mol Sci. 2025 Feb 15;26(4):1673. doi: 10.3390/ijms26041673 (PMC11854935; doi:10.3390/ijms26041673)
Supplement: Supplementary file 1 [file ijms-26-01673-s001.zip › Supplementary_File_S16.pdf]

Compound	Chemical Structure Depiction	PubChem ID#	Molecular Weight	Molecular Formula	PubChem Citation
Curcumin	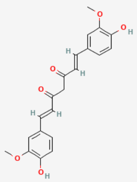	969516	368.4 g/mol	$C_{21}H_{20}O_6$	PubChem [Internet]. Bethesda (MD): National Library of Medicine (US), National Center for Biotechnology Information; 2004-. PubChem Compound Summary for CID 969516, Curcumin; [cited 2024 Nov. 3]. Available from: https://pubchem.ncbi.nlm.nih.gov/compound/Curcumin
Artemisinin	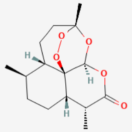	68827	282.33 g/mol	$C_{15}H_{22}O_5$	PubChem [Internet]. Bethesda (MD): National Library of Medicine (US), National Center for Biotechnology Information; 2004-. PubChem Compound Summary for CID 68827, Artemisinin; [cited 2024 Nov. 3]. Available from: https://pubchem.ncbi.nlm.nih.gov/compound/Artemisinin
Lutein	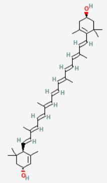	5281243	568.9 g/mol	$C_{40}H_{56}O_2$	PubChem [Internet]. Bethesda (MD): National Library of Medicine (US), National Center for Biotechnology Information; 2004-. PubChem Compound Summary for CID 5281243, Lutein; [cited 2024 Nov. 3]. Available from: https://pubchem.ncbi.nlm.nih.gov/compound/Lutein
5-Hydroxyflavone	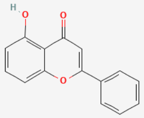	68112	238.24 g/mol	$C_{15}H_{10}O_3$	PubChem [Internet]. Bethesda (MD): National Library of Medicine (US), National Center for Biotechnology Information; 2004-. PubChem Compound Summary for CID 68112, 5-Hydroxyflavone; [cited 2024 Nov. 3]. Available from: https://pubchem.ncbi.nlm.nih.gov/compound/5-Hydroxyflavone
6-Hydroxyflavone	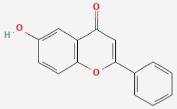	72279	238.24 g/mol	$C_{15}H_{10}O_3$	PubChem [Internet]. Bethesda (MD): National Library of Medicine (US), National Center for Biotechnology Information; 2004-. PubChem Compound Summary for CID 68112, 5-Hydroxyflavone; [cited 2024 Nov. 3]. Available from: https://pubchem.ncbi.nlm.nih.gov/compound/5-Hydroxyflavone
Zeaxanthin	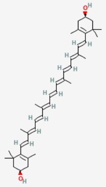	5280899	568.9 g/mol	$C_{40}H_{56}O_2$	PubChem [Internet]. Bethesda (MD): National Library of Medicine (US), National Center for Biotechnology Information; 2004-. PubChem Compound Summary for CID 5280899, Zeaxanthin; [cited 2024 Nov. 3]. Available from: https://pubchem.ncbi.nlm.nih.gov/compound/Zeaxanthin

Violaxanthin	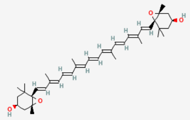	448438	600.9 g/mol	$C_{40}H_{56}O_4$	PubChem [Internet]. Bethesda (MD): National Library of Medicine (US), National Center for Biotechnology Information; 2004-. PubChem Compound Summary for CID 448438, Violaxanthin; [cited 2024 Nov. 3]. Available from: https://pubchem.ncbi.nlm.nih.gov/compound/Violaxanthin
Cerulenin	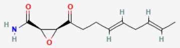	5282054	223.27 g/mol	$C_{12}H_{17}NO_3$	PubChem [Internet]. Bethesda (MD): National Library of Medicine (US), National Center for Biotechnology Information; 2004-. PubChem Compound Summary for CID 5282054, Cerulenin; [cited 2024 Nov. 3]. Available from: https://pubchem.ncbi.nlm.nih.gov/compound/Cerulenin
(-)-Epigallocatechin-3-Gallate (EGCG)	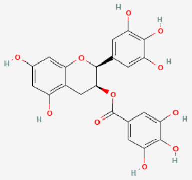	65064	458.4 g/mol	$C_{22}H_{18}O_{11}$	PubChem [Internet]. Bethesda (MD): National Library of Medicine (US), National Center for Biotechnology Information; 2004-. PubChem Compound Summary for CID 65064, Epigallocatechin Gallate; [cited 2024 Nov. 3]. Available from: https://pubchem.ncbi.nlm.nih.gov/compound/Epigallocatechin-Gallate
Cyano-myracrylamide (CMA)	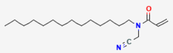	167312180	306.5 g/mol	$C_{19}H_{34}N_2O$	PubChem [Internet]. Bethesda (MD): National Library of Medicine (US), National Center for Biotechnology Information; 2004-. PubChem Compound Summary for CID 167312180, Cyano-myracrylamide; [cited 2024 Nov. 3]. Available from: https://pubchem.ncbi.nlm.nih.gov/compound/Cyano-myracrylamide
Benzosceptrin C	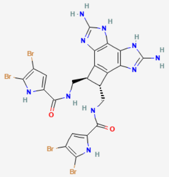	44598334	774.1 g/mol	$C_{22}H_{18}Br_4N_{10}O_2$	PubChem [Internet]. Bethesda (MD): National Library of Medicine (US), National Center for Biotechnology Information; 2004-. PubChem Compound Summary for CID 44598334, Benzosceptrin C; [cited 2024 Nov. 3]. Available from: https://pubchem.ncbi.nlm.nih.gov/compound/Benzosceptrin-C
2-(2-Hydroxy-5-nitro-benzylidene)-benzo[b]thiophen-3-one (Compound V)	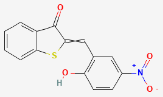	742147	299.3 g/mol	$C_{15}H_9NO_4S$	PubChem [Internet]. Bethesda (MD): National Library of Medicine (US), National Center for Biotechnology Information; 2004-. PubChem Compound Summary for CID 742147, 2-(2-Hydroxy-5-nitro-benzylidene)-benzo[b]thiophen-3-one; [cited 2024 Nov. 3]. Available from: https://pubchem.ncbi.nlm.nih.gov/compound/742147
