# Supplementary material for: zDHHC-Mediated S-Palmitoylation in Skin Health and Its Targeting as a Treatment Perspective
Source: Int J Mol Sci. 2025 Feb 15;26(4):1673. doi: 10.3390/ijms26041673 (PMC11854935; doi:10.3390/ijms26041673)
Supplement: Supplementary file 1 [file ijms-26-01673-s001.zip › Supplementary_File_S18.pdf]

## LEGEND FOR SUPPLEMENTARY FILES

**Supplementary File S1. List of Skin Diseases and their Treatments from STATPEARLS.** This figure shows a list of 14 skin conditions ordered alphabetically, and STATPEARLS was used to find a description of each of these conditions and their various treatments. The date STATSPEARLS was accessed is listed as well.

**Supplementary File S2. List of Search Terms Used in PubMed Regarding Skin Diseases and Skin Barrier Function Proteins.** This figure lists the search terms used in PubMed to find relevant articles regarding skin diseases and skin barrier function proteins. This figure is organized by the search term used and lists out the number of results obtained and relevant articles. The date of when the searches were performed is provided.

**Supplementary File S3. List of Search Terms Used in PubMed Regarding S-Palmitoylation, and the Trafficking and Signaling of zDHHC Enzymes.** This figure lists the search terms used in PubMed to find relevant articles regarding S-palmitoylation and the trafficking and signaling of zDHHC enzymes, and their relation to the skin. This figure is organized by the search term used and lists out the number of results obtained and relevant articles. The date of when the searches were performed is provided.

**Supplementary File S4. List of Search Terms Used in PubMed Regarding Phytochemicals and Their Influence on Palmitoylation.** This figure lists the search terms used in PubMed to find relevant articles regarding phytochemicals and their influence on palmitoylation. This figure is organized by the search term used and lists out the number of results obtained and relevant articles. The date of when the searches were performed is provided.

**Supplementary File S5. List of Search Terms Used in PubMed Regarding the Relation of Palmitoylation to Skin and Specific Skin Diseases.** This figure lists the search terms and search results gathered from PubMed regarding the of palmitoylation to the skin and skin diseases. This figure is organized by the search term used and lists out the number of hits from the search and the number of relevant articles. A final article PMID list was also generated in numerical order with any replicate articles removed. Dates for when the searches were conducted are provided.

**Supplementary File S6. List of Search Terms Used in PubMed for Skin Barrier Protein.** This figure lists the skin barrier proteins with their associated functions. This figure is organized by the search term used with the number of hits and relevant articles. The date of when the searches were performed are provided.

**Supplementary File S7. List of Search Terms Used in PubMed regarding Palmitoylation in ErbB and PLSCR Protein Families.** This figure lists the PubMed search terms utilized to find data on the palmitoylation status of the ErbB and PLSCR protein families and their relations to skin. This figure is organized by the search term used and the number of hits and relevant PubMed articles for each term.

**Supplementary File S8. cBioportal Data Sets for Skin Barrier Proteins.** cBioportal results for 47 skin barrier function proteins are shown in each worksheet labeled as "cancer_types_summary". The cancer study name, alteration frequency, alteration type, and alteration count are displayed on each of these worksheets. Moreover, there is an additional 47 worksheets labeled as "Updated", in which details of the methods are listed in Column B and without the Skin Cutaneous Melanoma (TCGA, PanCancer Atlas) dataset.

**Supplementary File S9. cBioportal Data Sets for ErbB Family Members.** cBioportal results for 4 ErbB family members (ErbB1/EGFR, ErbB2, ErbB3, and ErbB4) are shown in each worksheet labeled as "cancer_types_summary". The cancer study name, alteration frequency, alteration type, and alteration count are displayed on each of these worksheets. Moreover, there is an additional 4 worksheets labeled as "Updated", in which details of the methods are listed in Column B and without the Skin Cutaneous Melanoma (TCGA, PanCancer Atlas) dataset.

**Supplementary File S10. cBioportal Data Sets for Phospholipid Scramblase (PLSCR) Members.** cBioportal results for 7 PLSCR family members (PLSCR1, PLSCR2, PLSCR3, PLSCR4, PLSCR5, TMEM16F, and XKR8) are shown in each worksheet labeled as "cancer_types_summary". The cancer study name, alteration frequency, alteration type, and alteration count are displayed on each of these worksheets. Moreover, there is an additional 7 worksheets labeled as "Updated", in which details of the methods are listed in Column B and without the Skin Cutaneous Melanoma (TCGA, PanCancer Atlas) dataset.

**Supplementary File S11. Characteristics of Human zDHHC Enzymes.** This figure lists UniProt ID#, UniProt protein size (amino acids and Da), HGNC ID#, and HGNC alias symbols.

**Supplementary File S12. Methodologies Applied to Assay Palmitoylation.** The figure illustrates the respective methods used to assay palmitoylation with respect to the PMID# listed. The most common methods utilized include Acyl-Biotin Exchange (ABE), metabolic labeling, and click chemistry. The PMIDs are organized in chronological order from 1996 to 2024.

**Supplementary File S13. Evidence-Based Determination of Palmitoylation Status for a Subset of Skin Barrier Function Proteins.** The figure lists the different skin barrier proteins, organized by PMID# and the date of publication. This figure identifies the protein, the organism, and the cell system in which the protein was studied. This figure highlights the palmitoylation methodology, to decipher the palmitoylation status and the prediction program that was applied to predict specific sites.

**Supplementary File S14. Evidence-Based Determination of Palmitoylation Sites for ErbB and Scramblase Family.** The figure lists the different experimental methodologies utilized to identify the palmitoylation sites across an array of mammalian cell lines, organized by PMID# and the date of publication. The figure identifies the ErbB or scramblase family member, the organism, and the cell system in which the protein was studied. This figure highlights the palmitoylation methodology, the palmitoylation status, and the prediction program that was applied to predict specific sites.

**Supplementary File S15. Experiments with 2-Bromopalmitate (2-BP) and their Cellular/Functional Outcomes.** This figure includes a list of articles, organized in ascending order by PMID #, that used 2-bromopalmitate, its doses, its treatment times, their cell lines and model systems, the assessments used, and the outcomes of their experiments.

**Supplementary File S16. Structures and Characteristics of Palmitoylation Inhibitors.** Chemical structures were obtained from PubChem [192], along with their molecular weights, ID #, molecular formula, and date updated.

**Supplementary File S17. Localization of Tagged ZDHHCs across an Array of Mammalian Cell Lines.** This figure organizes the findings of relevant primary research articles according to their PMID# and date of publication. Key details include the experimental model system [mammalian cell lines], the tagged protein being overexpressed, and the methodology utilized. Please note that there is a lack of studies which attempt to determine the localization of endogenous proteins. Please note that the information extracted from PMID# 16647879 has been converted from DHHC to zDHHC nomenclature.
